# Gender representation begets gender representation

**DOI:** 10.1371/journal.pone.0351865

**Published:** 2026-08-26

**Authors:** Stella F. Lourenco, Kylee Novick, Ryno Kruger, Arber Tasimi

**Affiliations:** Department of Psychology, Emory University, Atlanta, Georgia; University of Washington Bothell, UNITED STATES MINOR OUTLYING ISLANDS

## Abstract

Research on the underrepresentation of women in academia and other professions indicates that women may be underrepresented in fields associated with intrinsic aptitude—or “brilliance”—because women are stereotyped as not possessing brilliance. At the same time, women may be less likely to pursue careers in such fields because few women are in them, which highlights the importance of understanding the role of gender representation in guiding professional interests. Across four studies (*N*  = 1,680), we found that people have knowledge of the male-female breakdown of employees for a broad range of occupations (Study 1) and that current gender demographics predicts interest and belonging in hypothetical advertised employment (Studies 2–4). The effect of gender demographics was robust, even prioritized over messaging about ability, particularly among women (Study 3), and scaling parametrically according to the ratio of male and female employees (Study 4). Especially among women, professions with more females elicited greater interest and feelings of belonging than those with more males, even when those professions were associated with brilliance. These findings suggest that altering beliefs about brilliance for success in specific professions, though important, may be insufficient for increasing female representation in fields where women are underrepresented. Instead, increasing future female representation may benefit from increasing the presence of more women now.

## Introduction

Half of the United States population is female, yet women remain in the minority in crucial sectors such as politics [1], technology [2], science [3,4], and academia [5,6]. These inequities have tangible impacts on women’s societal status, economic livelihood, and overall well-being [7]. What accounts for the underrepresentation of women, and why has it persisted?

One explanation centers on culturally ambient stereotypes that link men, but not women, with “brilliance.” If so, women may be considered unfit, or feel unwelcome, in fields believed to emphasize brilliance. The idea that female underrepresentation may reflect the level of brilliance, or intrinsic aptitude, associated with that field is at the heart of the Field-Specific Ability Beliefs (FAB) model ([8]; see also, [9–12]). Consistent with the FAB model, there is a negative relation between academics’ beliefs about the brilliance required to succeed in their field and the corresponding percentage of PhDs received by women. For example, fields like physics and philosophy emphasize brilliance more than fields like psychology and education, and fewer women earn PhDs in physics and philosophy compared to psychology and education [8,10]. Even in experimental contexts, exposure to a hypothetical college major, internship, or job that ostensibly necessitates brilliance leads women, but not men, to report less interest in pursuing those opportunities [13].

When considering the FAB model, however, it can be difficult to disentangle beliefs about the brilliance required for a specific profession from the *actual* gender representation in that profession. For example, if women are underrepresented in a field considered to require brilliance, are they less likely to pursue a career in that field because of its emphasis on brilliance, or because they expect there to be fewer women? Research over the last decade has largely focused on the former possibility, with growing consensus that the greater the premium placed on brilliance within a field, the less likely women are to show interest in it.

Less attention has been paid to the other possibility—that current gender representation begets future gender representation—despite evidence indicating that people are aware of gender disparities in the everyday world. In one study, for example, participants estimated how many women had received doctoral degrees in academic fields [10]. FAB remained predictive of gender representation when accounting for participants’ estimates of the actual gender distribution, but only among participants with a college degree. Among those without college degrees, FAB did not predict actual gender representation when accounting for participants’ expectations about the male-female distribution in various fields. Other research has demonstrated that knowledge of gender demographics affects women’s interest in jobs [14], anticipated belonging at conferences [15], and impressions of occupational climates more generally [16].

Nevertheless, it remains unclear how people’s awareness of gender demographics influences their interest and anticipated sense of belonging across professions within and beyond the academy. Although proponents of the FAB model acknowledge the importance of current gender representation in predicting future gender representation [8], no research, to our knowledge, has directly examined how gender demographics are weighted relative to FAB when considering the future representation of women across professional fields. The present research therefore draws on relational-demography and social-identity perspectives (Cejka & Eagly, 1999; Koenig & Eagly, 2014), which posit that people are motivated to affiliate with groups in which they perceive in-group representation, a form of gender-based homophily that enhances anticipated belonging and approach motivation. In this respect, gender ratios may serve as a salient cue to social fit, signaling whether one’s values and behavioral norms will be shared, over and above an emphasis on brilliance.

Researchers have attempted to account for the FAB model’s specificity by assessing whether estimated gender distribution (e.g., whether people expect a field to be predominantly male or female) can explain people’s beliefs about the brilliance required for an occupation. This work provides some support for a dissociation between perceived gender distributions and FAB [8], but it does not rule out that current representation plays a role in future representation, over and above that of FAB. In other words, although FAB may not be rooted in our knowledge of gender demographics, such knowledge may nevertheless shape interest and belonging in ways that sustain gender gaps.

What is more, research by Vial and colleagues [17] demonstrated that expected gender representation for an occupation predicted people’s perceptions of workplace culture, specifically a “masculinity contest culture” (MCC) that emphasizes dominance and competition. They also found that expected gender representation remained a significant predictor of participants’ interest and anticipated belonging for a hypothetical job, even after accounting for FAB. This study, however, did not experimentally manipulate FAB, nor gender demographics. Thus, it remains unknown how people weight information about current gender representation relative to FAB when considering future employment.

### Present research

Here, we sought to understand whether people’s expectations of gender demographics work to sustain existing gender disparities by influencing their interest and anticipated sense of belonging across a variety of actual and hypothetical jobs. We also aimed to clarify how perceived gender representation might interact with, and perhaps even override, the influence of FAB. In addition to FAB, recent research has pointed to other variables related to workplace fit that may shape interest and belonging in the workplace. These variables include MCC [18,19], as previously mentioned, and prototype matching (PM), which reflects the alignment between oneself and representative group members [20,21]. MCC has even been found to mediate the effect of FAB in people’s job interest [17]. Specifically, fields that emphasize dedication rather than brilliance tend to be less associated with MCC, thereby increasing interest and/or anticipated belonging, especially among women. We therefore examined these variables alongside FAB and gender representation. MCC and PM were included as theoretically relevant covariates rather than mediators, given our focus on establishing the independent predictive value of gender representation.

Study 1 provided a first test of the role of perceived gender representation in sustaining gender disparities in employment by assessing whether participants from the general population are sensitive to the distribution of men and women across a variety of occupations. We also examined the extent to which participants’ estimates of gender representation predicted belonging in these various real-world occupations. Studies 2–4 then provided causal tests of the relevance of perceived gender representation to future representation. In these studies, participants were presented with hypothetical job advertisements that emphasized the importance of either brilliance or dedication, after which participants rated their interest and anticipated belonging for these jobs. In Study 2, we asked participants to estimate the distribution of men and women in each job. In Study 3, we instead provided participants with explicit information about the male-female distribution in each job, which, importantly, sometimes involved a conflict between a gender stereotype based on brilliance and the gender representation for that job (e.g., a job ad emphasizing brilliance with a larger proportion of female employees). Finally, in Study 4, we directly manipulated the ratio of male-to-female representation, which allowed us to examine whether people’s interest and belonging scaled parametrically with the distribution of employees who shared their gender identity. Altogether, these studies provide a comprehensive test of whether and how gender demographics influence interest and belonging—and, by extension, the future representation of women across professional fields.

### Transparency and openness

All studies were approved by the Institutional Review Board (IRB) at Emory University. Studies 1–3 were not preregistered. Study 4’s design and statistical analyses were preregistered at AsPredicted.org (https://aspredicted.org/n82x-6rfx.pdf). Sample size determinations, data exclusions, manipulations, and measures across studies are all reported. Studies follow JARS [Appelbaum et al., 2018]. All data are uploaded on Open Science Framework (OSF) (https://osf.io/23e5h).

## Study 1

If current gender demographics were to influence future representation, then people would need to have knowledge of the male-female distributions of various occupations. Using a broad sample of real-world occupations, Study 1 assessed participants’ knowledge of gender representation across the workforce, and how such knowledge might influence their sense of belonging across occupations in the everyday world. We predicted that if gender demographics affect future representation, then participants’ anticipated belonging should be related to their estimates of the male-female demographics. Following previous research (e.g., [17]), we also considered people’s perceptions of FAB and workplace fit to assess the specificity of these different factors and how they might jointly contribute to future gender representation.

### Method

#### Participants.

A total of 103 participants were recruited through the Prolific online testing platform. We focused exclusively on participants identifying as male or female, which is currently the norm within the extant literature on this topic. Thus, seven participants were excluded from analyses because they did not identify as male or female. The remaining sample in this study (*N* = 96; *M*_*age*_ = 30.7 years, range = 18–64 years; 60 women, 36 men) exceeded that determined by an a priori power analysis conducted with G*Power 3.1 (*N* = 84, α = .05, 1 – *β* = .80, moderate-to-large effect size, *f*^*2*^ = 0.135). A sensitivity analysis confirmed that this sample was powered to detect a minimum correlation of *r* = .315. All participants were located in the United States and compensated $11/hour.

#### Procedure.

**Fig 1:**
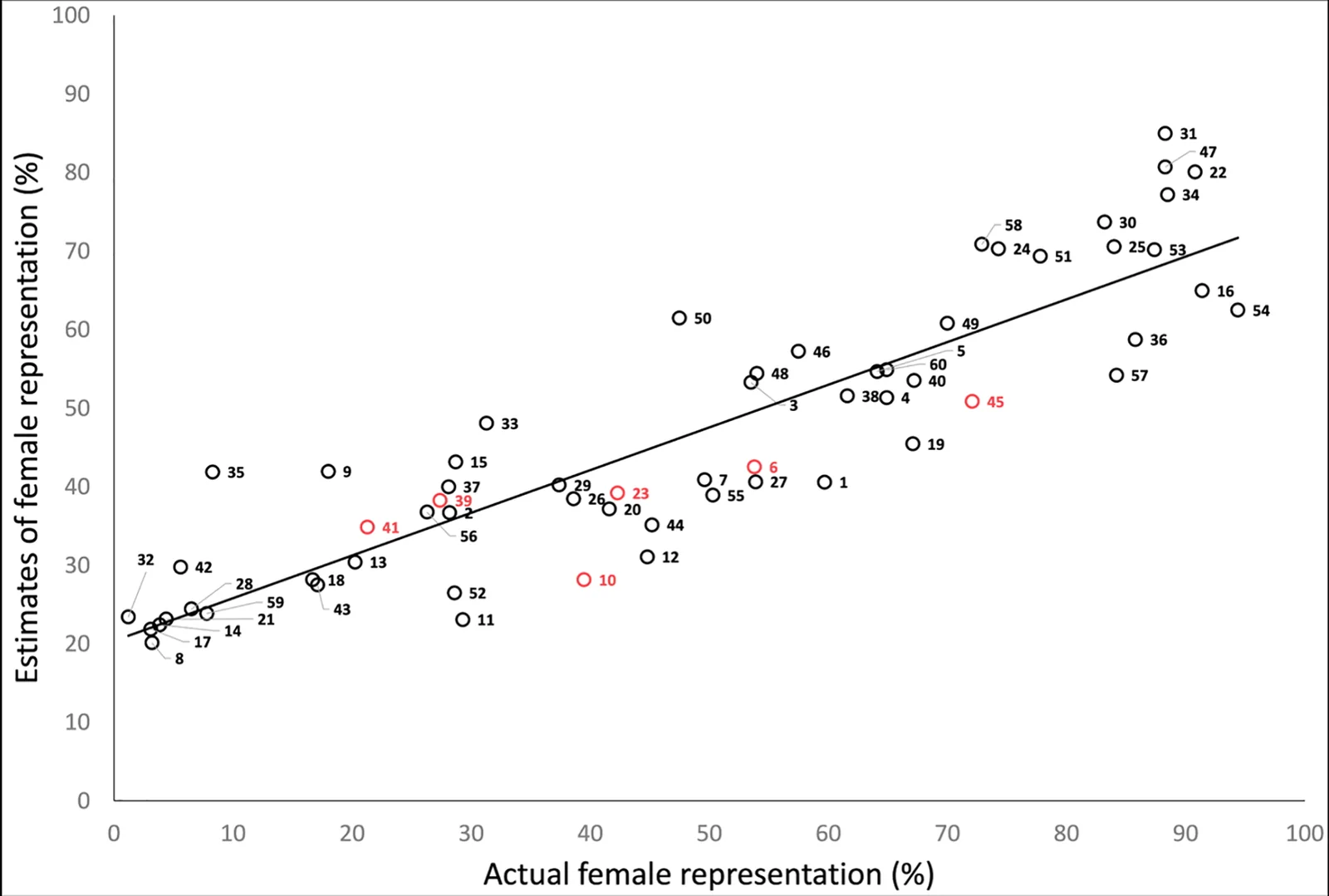
Scatterplot of the correlation between estimated and actual gender representation in Study 1. *Note.* Red markers indicate academic professions. Black markers indicate non-academic professions. The full list of 60 occupations included in this study appears below: 1: Accountants and Auditors; 2: Architects; 3: Artists; 4: Authors; 5: Bakers; 6: Biology Professors; 7: Bus Drivers; 8: Carpenters; 9: Chefs; 10: Chemistry Professors; 11: Chief Executives (i.e., CEOs); 12: Coaches; 13: Computer Programmers and Software Developers; 14: Construction Workers; 15: Dentists; 16: Dieticians and Nutritionists; 17: Electricians; 18: Engineers; 19: Exercise/Fitness Trainers; 20: Financial and Investment Analysts; 21: Firefighters; 22: Hairdressers; 23: History Professors; 24: Human Resources Personnel; 25: Interior Designers; 26: Janitors and Cleaning Staff; 27: Judges; 28: Landscapers; 29: Lawyers; 30: Librarians; 31: Maids/Housekeepers; 32: Mechanics; 33: Musicians; 34: Nurses; 35: Painters; 36: Paralegals and Legal Assistants; 37: Paramedics; 38: Pharmacists; 39: Philosophy Professors; 40: Physical Therapists; 41: Physics Professors; 42: Pilots; 43: Police Officers; 44: Producers and Directors; 45: Psychology Professors; 46: Real Estate Agents; 47: Receptionists; 48: Reporters and Journalists; 49: Restaurant Servers; 50: Retail Workers; 51: School Counselors; 52: Security Guards; 53: Social Workers; 54: Speech-Language Pathologists; 55: Statisticians; 56: Surgeons; 57: Tailors; 58: Teachers; 59: Truck Drivers; 60: Veterinarians.

Participants were presented with an occupation and asked to estimate the gender ratio within that occupation using a sliding scale anchored by 0% (all men) and 100% (all women). Each participant was assigned 30 occupations (in randomized order) out of a total of 60 (see Fig 1 for the full list). Each subset included 27 non-academic jobs selected from the U.S. Bureau of Labor Statistics (2021) [22] database and three academic jobs, converted from fields into jobs (e.g., “biology” to “biology professor”). The two subsets of occupations were consistent in overall average gender representation.

After estimating gender representation for all occupations, participants completed four separate measures (fixed order) designed to assess the constructs of interest: *FAB* [10], *belonging* [13], *MCC* [23], and *PM* [13]. The FAB questionnaire included four items (e.g., “Being a [] requires a special aptitude that just can’t be taught.”) rated on a scale of 1–7 from “strongly disagree” to “strongly agree.” Two belonging items (e.g., “With the appropriate training and resources, I feel like I would belong in this occupation.”) were rated on the same scale. The MCC questionnaire included three items (e.g., “In this job, one person’s loss is another person’s gain.”) rated on a 1−5 scale from “strongly disagree” to “strongly agree.” Finally, PM was assessed with a single item (“How similar do you think you are to the other people who hold this occupation?”), rated on a 1−7 scale from “not at all similar” to “very similar.” (See Supporting Information [S1 File] for all items.) As in previous research [13,17], composite scores were computed as the mean of the items for each multi-item measure.

### Results

#### How accurate are participants’ estimates of gender representation?

A zero-order correlation at the occupation level revealed that participants’ average estimates were significantly correlated with actual gender representation, *r*(58) =.891, *p* < .001 (see Fig 1). This relation held across both academic (*r*[4] =.932) and non-academic (*r*[52] =.896) occupations, with no significant difference between them (Fisher’s z = 0.378, *p* = .705); both correlations were statistically significant (*p*s < .001).

We next asked whether participants’ sensitivity to gender representation depended on their education level or gender. To do so, we analyzed the data at the trial level (i.e., individual estimates rather than occupation-level averages), controlling for participant gender and education. Even when accounting for these factors, estimates remained significantly correlated with actual gender representation, *r*_*p*_(2830) =.639, *p* < .001. Overall, these results demonstrate that people are sensitive to real-world male-female distributions across a variety of occupations. Although participants saw only a subset of occupations, the strong correspondence at the group level suggests broad and accurate knowledge of gender demographics.

#### Is perceived gender representation related to anticipated belonging?

Having found that participants’ estimates of gender representation track with reality, we next examined whether perceived representation predicted anticipated belonging for different occupations, when also considering the potential effects of FAB, MCC, and PM. Trial-level multiple regression analyses using participants’ estimates and ratings revealed that all predictors were significant (model *R*^*2*^ = .306, *f*^*2*^ = .441): estimated gender representation (*β* = 0.108, *t* = 6.75, *p* < .001), FAB (*β* = −0.231, *t* = −14.73, *p* < .001), MCC (*β* = −0.225, *t* = −14.08, *p* < .001), and PM (*β* = 0.444, *t* = 27.51, *p* < .001). Following previous s*t*udies, we used a composite score for the belonging measure; however, the two items were not significantly correlated with one another. Thus, effects of FAB, MCC, and PM were also examined separately by item (results were largely consistent between the two items [see S2 File]).

Given previous findings suggesting that such effects may vary by participant gender (e.g., [13]), we conducted an additional regression analysis including participant gender and its two-way interactions with the other variables (model *R*^*2*^ = .328, *f*^*2*^ = .488). Participant gender was dummy coded (female = 1, male = 0). There were significant interactions between participant gender and estimates of female representation (*β* = 0.138, *t* = 4.22, *p* < .001) and between participant gender and PM (*β* = 0.192, *t* = 5.77, *p* < .001). Simple effects were examined to clarify the direction of these interactions (see S2 File). Analyses revealed that whereas women’s belonging was influenced by their estimates of male-female demographics (i.e., more female employees, greater belonging), men’s belonging was not. PM ratings were significantly related to belonging (i.e., greater PM, greater belonging), though somewhat more strongly for women than men. Participant gender did not interact with MCC (*β* = −0.025, *t* = −0.77, *p* = .441), nor with FAB (*β* = −7.64 × 10^−4^, *t* = −0.02, *p* = .981), when belonging was measured as a composite (see S2 File for FAB by participant gender interaction with individual items).

Overall, these findings largely replicate previous research suggesting that beliefs about brilliance [13,23,24] and workplace fit [25–27] contribute to people’s sense of belonging for different occupations. Critically, they extend this work by also demonstrating the importance of perceived gender demographics, particularly among women. The greater the expected female representation, the higher women’s anticipated belonging, even when accounting for effects of FAB, MCC, and PM.

## Study 2

In a second study, we more directly probed the impact of people’s expectations of gender demographics and FAB on appraisals of future employment. Participants were presented with hypothetical job advertisements that either emphasized the need for brilliance or dedication of its employees, following previous work [13]. They then rated their interest in applying for the position and their anticipated sense of belonging were they to be employed there. In addition, participants estimated the gender representation of each job. By comparing appraisals for jobs emphasizing brilliance versus dedication considering the expected male-female representation, we tested whether appraisals of occupational fit are driven more by beliefs about required ability (i.e., FAB) or by its assumed gender (im)balance (i.e., male-female demographics).

### Method

#### Participants.

A total of 213 participants were recruited through Prolific. Of these, 19 were excluded from analyses for failing the manipulation check (*n* = 13) or for not identifying as male or female (*n* = 6). The remaining sample comprised 194 participants (*M*_*age*_ = 36.3 years, range = 18–74 years; 95 women, 99 men). Given the similarity of our procedures to those used in previous research [13], we targeted a comparable sample size, which exceeded the requirement for adequate power (*α* = .05, 1 – *β* = .80). A sensitivity analysis (G*Power 3.1) indicated power to detect effects ranging from *f²* = 0.057–0.109, corresponding to small-to-medium magnitudes [Cohen, 1988]. Eligibility criteria (U.S.-based; 18 + years of age) and compensation rate matched Study 1. Participants from Study 1 were ineligible.

#### Procedure.

Participants were randomly assigned to view one of two job advertisements (adapted from [13]), ostensibly posted on “a popular job website.” Ads emphasized either brilliance (*n* = 101) or dedication (*n* = 93) as needed to succeed in the position and edited to match in word length. Phrases emphasizing brilliance included: “high IQ, superior reasoning skills, and a knack for big, bold ideas;” “intellectual abilities [that] stand out;” and “natural intelligence.” Phrases emphasizing dedication included: “strong work ethic and a commitment to completing…work well;” “sustained dedication in…past positions;” and “consistent effort to achieve goals.” See S1 File for full job descriptions. Participants were instructed to imagine that they had “been out of a job for a couple of weeks and [were] looking for a new job.” They then provided ratings of interest and anticipated belonging, as indices of potential future representation. (Note that the interest measure was not included in Study 1 because the occupations were real and, thus, would likely be confounded with personal preferences and individual perceptions of each occupation.) As in the previous study, participants also rated MCC and PM; they were also asked to estimate the gender distribution of the employees.

Two questions gauged participants’ interest in the job opportunity (e.g., “Assuming you were looking for a job, how likely would you be to apply for this particular job?”; 1 = “not at all likely” to 9 = “extremely likely;” see S1 File)**.** Anticipated belonging was measured with two questions suited to the hypothetical context: “I would feel like I belong” and “I would feel like I always have to prove myself” (reverse-coded). These differed slightly from the belonging items used in Study 1, which referenced real occupations. MCC [23] and PM [13] were assessed as in Study 1, with an additional PM item capturing job-requirement fit (“How well do you think you match the requirements for this job?”; 1 = “not at all” to 9 = “very much” [28]). Finally, like Study 1, participants estimated gender representation (sliding scale anchored by 0% [all men] and 100% [all women]). Measures were presented in randomized order, except for the gender estimation, which participants either answered before or after the other measures (randomized). As a manipulation check, all participants were asked whether the advertisement they read emphasized “intellectual ability” or “sustained dedication” after completing all other measures.

### Results

Items within measure were significantly correlated: interest (*r*[192] =.849, *p* < .001), belonging (*r*[192] =.254, *p* < .001), MCC (*rs*[192] >.634, *ps* < .001) and PM (*r*[192] =.684, *p* < .001). Cronbach’s alphas were high for measures of interest (0.918), MCC (0.852), and PM (0.812), but lower for belonging (0.405). Composite scores were used in subsequent analyses, including for the belonging measure with modest reliability to ensure comparability with prior research [13]. Nevertheless, separate analyses for each item in the belonging measure were conducted for all studies (see SI).

Like Study 1, participants were asked to estimate the male-to-female ratio of employees per job. There was no overall difference in estimated gender ratio between the hypothetical job ads, *t*(192) = −0.387, *p* = .699, *d* = −0.056. Participants estimated lower female representation for both jobs (brilliance ad: *M* = 39.1% women, *SD* = 16.8; dedication ad: *M* = 40% women, *SD* = 14.8). Nevertheless, the individual differences in estimates allowed us to test the extent to which participants’ expectations of the gender representation affected their interest and anticipated belonging for each job.

#### Does FAB messaging affect interest and anticipated belonging?

**Fig 2:**
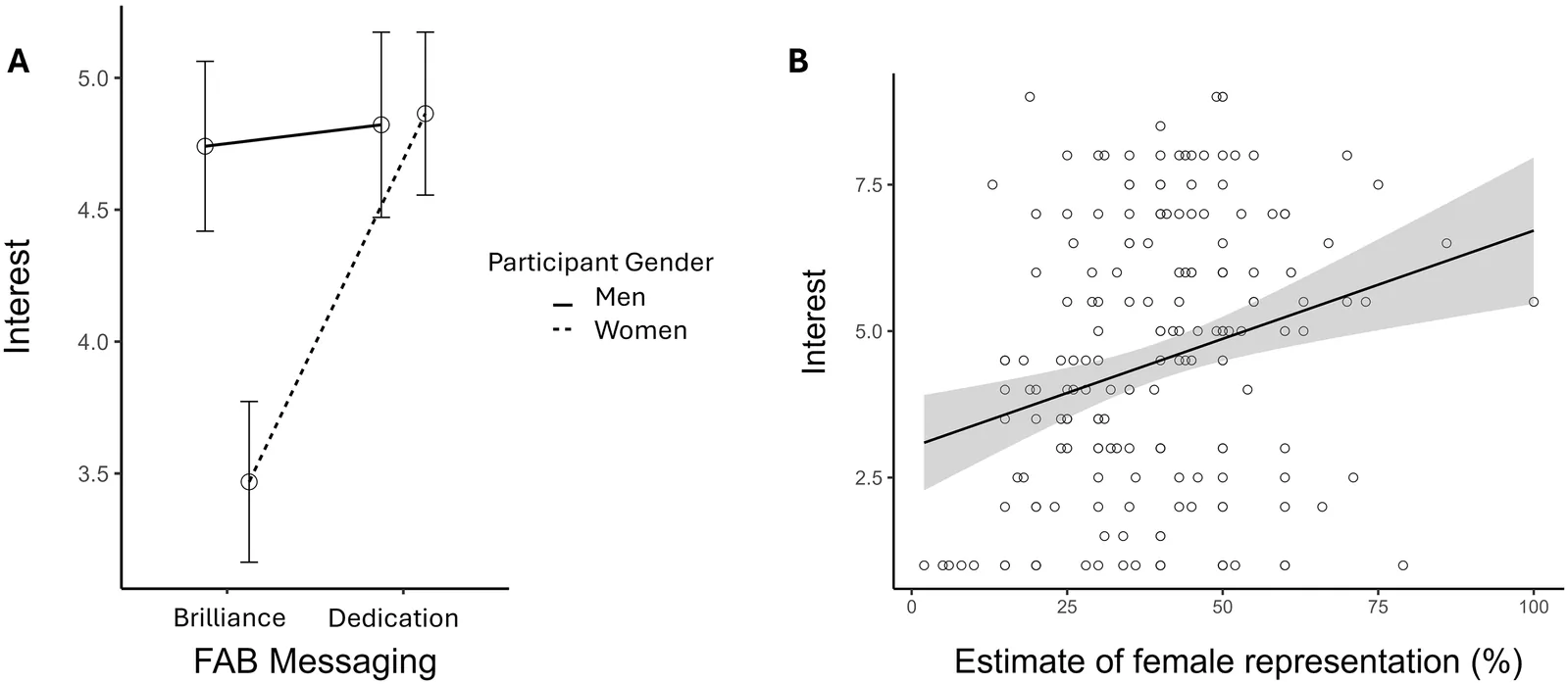
Participants’ ratings of interest for the advertised jobs in Study 2. *Note.* Interest ratings (average of 1-9 ratings for two items) plotted as a function of FAB messaging and gender (A), and across expected gender representation (B). Error bars represent standard error (SE); horizontal jitter was added to enhance visual discrimination (A). The shaded region on the scatterplot (B) represents the 95% confidence interval (CI).

We first tested for effects of FAB on participants’ ratings of job interest and anticipated belonging if employed there, as a replication of previous research. A multiple regression analysis, with FAB messaging (dedication = 1, brilliance = 0), participant gender (female = 1, male = 0), and their interaction as predictors of interest ratings, revealed a significant main effect of participant gender, as well as an interaction between FAB messaging and participant gender (Table 1). Given the significant interaction, follow-up gender-specific regressions were also conducted (see S3 File). Analyses revealed that FAB messaging showed a statistically reliable effect for women on their ratings of interest, but not for men (see also Fig 2A). A separate multiple regression analysis on anticipated belonging yielded no main effects or interactions (see S3 File). Nevertheless, to parallel analyses on interest, we conducted additional gender-specific regressions, which revealed that women showed a significant effect of FAB messaging (greater belonging for dedication than brilliance), but men did not (see S3 File). These results are generally consistent with previous findings suggesting that women, in particular, express greater interest and belonging in jobs emphasizing dedication over brilliance (e.g., [13]).

**Table 1:** Study 2 regression models predicting participants’ interest in the advertised jobs.

Predictor	Model 1 (*R*^*2*^ = .063, *f*^*2*^ = .067)			Model 2 (*R*^*2*^ = .122, *f*^*2*^ = .139)			Model 3 (*R*^*2*^ = .567, *f*^*2*^ = 1.309)		
	*β*	*t*	*p*	*β*	*t*	*p*	*β*	*t*	*p*
FAB Messaging (Dedication [1 = dedication])	0.035	0.180	.857	0.068	0.352	.725	−0.209	−1.517	.131
Participant Gender (Female [1 = female])	−0.553	−2.843	**.005**	−0.519	−0.821	.413	−0.425	0.083	.934
FAB Messaging × Participant Gender	0.572	2.036	**.043**	0.494	1.800	.074	0.363	1.872	.063
Representation Estimates				0.271	3.080	**.002**	0.153	2.406	**.017**
Representation Estimates × Participant Gender				−0.079	−0.560	.576	−0.179	−1.802	.073
MCC							−0.215	−4.015	**<.001**
PM							0.609	11.606	**<.001**

*Note.* Standardized *β* coefficients are reported for all variables. Reference groups are Brilliance (FAB Messaging) and Male (Participant Gender).

#### Does expected gender representation affect interest and belonging?

In another set of analyses, we tested for potential effects of participants’ estimates of gender representation over and above that of FAB messaging. We compared a second multiple regression model (Model 2) with participants’ gender estimates to the aforementioned model (Model 1; see Table 1). The second model predicted significantly more variance than the first model for both ratings of interest (*ΔR^2^* = .059, *p* = .002) and belonging (*ΔR^2^* = .068, *p* = .001). Moreover, in the second model, only expected gender representation significantly predicted interest (see Table 1, see also, Fig 2B), which suggests that gender representation overrides the effect of FAB on job interest. Similarly, for belonging, only expected gender representation was significant (*β* = 0.186, *t* = 2.079, *p* = .039); no other predictors, nor interactions, reached significance (see S3 File). Participants expressed more interest, and greater anticipated belonging, when they estimated more women employees, regardless of whether the job ad emphasized brilliance or dedication.

Having found that estimated gender representation affects interest and anticipated belonging, we next tested whether MCC and PM also explained participants’ responses. In a third model, we included MCC and PM as predictors (see Table 1). We found that MCC and PM predicted both participants’ interest (MCC: *β* = −0.215, *t* = −4.015, *p* < .001; PM: *β* = 0.609, *t* = 11.606, *p* < .001; model *R*^*2*^ = .567, *f*^*2*^ = 1.309) and belonging (MCC: *β* = −0.400, *t* = −6.898, *p* < .001; PM: *β* = 0.430, *t* = 7.581, *p* < .001; model *R*^*2*^ = .494, *f*^*2*^ = .976). Moreover, when accounting for MCC and PM, estimated gender representation remained significant as a predictor of interest (*β* = 0.153, *t* = 2.406, *p* = .017); additional gender-specific regressions revealed that, interestingly, the effect was only significant in men, not women (see S3 File). This gender difference should be interpreted cautiously given that the interaction between participant gender and estimated gender representation was not statistically significant (Table 1). Estimated gender representation did not remain a significant predictor of anticipated belonging when accounting for MCC and PM (*β* = 0.036, *t* = 0.520, *p* = .604; see S3 File for gender-specific regressions and item-level analyses).

Altogether, the findings from Study 2 show effects of participants’ expected gender representation over and above that of FAB messaging. They also reveal important roles for MCC and PM in understanding how expected gender representation predicts interest and anticipated belonging in future employment.

## Study 3

Study 3 provided a more stringent test of whether gender representation influences people’s appraisals of workplace fit above and beyond FAB. As in Study 2, job advertisements emphasized either brilliance or dedication as the key to success. Here, however, each ad also specified a gender ratio among employees: predominantly male versus predominantly female. By manipulating both FAB messaging *and* gender representation, we directly tested whether people prioritize ability messaging or gender composition when evaluating their interest in pursuing an employment opportunity and their anticipated belonging once employed there.

### Method

#### Participants.

A total of 474 participants were recruited through Prolific. Of these, 75 were excluded from analyses for failing manipulation checks (*n* = 56) or not identifying as male or female (*n* = 19), leaving a final sample of 399 participants (*M*_*age*_ = 33.5 years, range = 18–85 years; 200 women, 199 men). Given four between-subjects conditions (compared to two in Study 2), the target sample size was doubled from the previous study. Eligibility criteria and compensation matched prior studies.

A sensitivity analysis (G*Power 3.1) indicated that, with *N* = 399, *α* = .05, a 2 (FAB messaging) × 2 (gender representation) × 2 (participant gender) ANOVA design provided power (.80) to detect an effect size of *f²* = 0.166, which is considered large by conventional standards [Cohen, 1988]. This threshold accounts for reduced power when testing multiple between-subjects effects and interactions [29].

#### Procedure.

Participants were randomly assigned to one of four experimental groups, which crossed the conditions of FAB messaging (brilliance vs. dedication) and gender representation (majority male vs. majority female). FAB messaging was identical to Study 2 in that participants were presented with advertisements that emphasized either brilliance or dedication. The gender representation condition manipulated whether the job ad reported a greater proportion of male or female employees (75% male vs. 75% female). Participants were again instructed to imagine being out of a job. After reading the job description, participants were told that the company “want[ed] to provide [them] with some information about our current employees.” Three of the points were conventional (i.e., information about retirement age, work week requirements, and benefits; see S1 File). The last item in the list provided information about the gender representation: either “75% of our employees are men and 25% are women” (male-dominated) or “75% of our employees are women and 25% are men” (female-dominated). After reading the ad description, participants responded to the following measures: interest, belonging, MCC, and PM.

All measures were randomly presented and identical to those in Study 2. Participants then completed two attention check questions (after the other measures) to ensure that the FAB messaging and gender representation manipulations were properly encoded. The first question was identical to that in Study 2 (“Did this advertisement emphasize intellectual ability or sustained dedication?”), and the second question asked participants to recall whether the job employed “mostly men” or “mostly women.”

### Results

Individual items were significantly correlated for each measure: interest (*r*[397] =.829, *p* < .001), belonging (*r*[397] =.185, *p* < .001), MCC (*r*s[396] >.571, *p*s < .001) and PM (*r*[397] =.600, *p* < .001). Cronbach’s alphas were high for measures of interest (0.906), MCC (0.827), and PM (0.749), and modest for belonging (0.313). We used composite scores for each measure in subsequent analyses (see S4 File for belonging item analyses).

Because Study 3 employed a fully crossed experimental design (FAB messaging × gender representation × participant gender), we analyzed the data using ANOVA/ANCOVA to estimate condition-level differences and interactions. This approach differs from Studies 1 and 2, which relied on correlational and regression analyses due to the use of continuous predictors. (For completeness, equivalent regression models are reported in the Supplemental Information; these models yield the same pattern of higher-order effects, with minor differences in lower-order terms reflecting differences in parameterization [e.g., reference group coding]).

#### How do FAB and actual gender representation affect interest?

**Fig 3:**
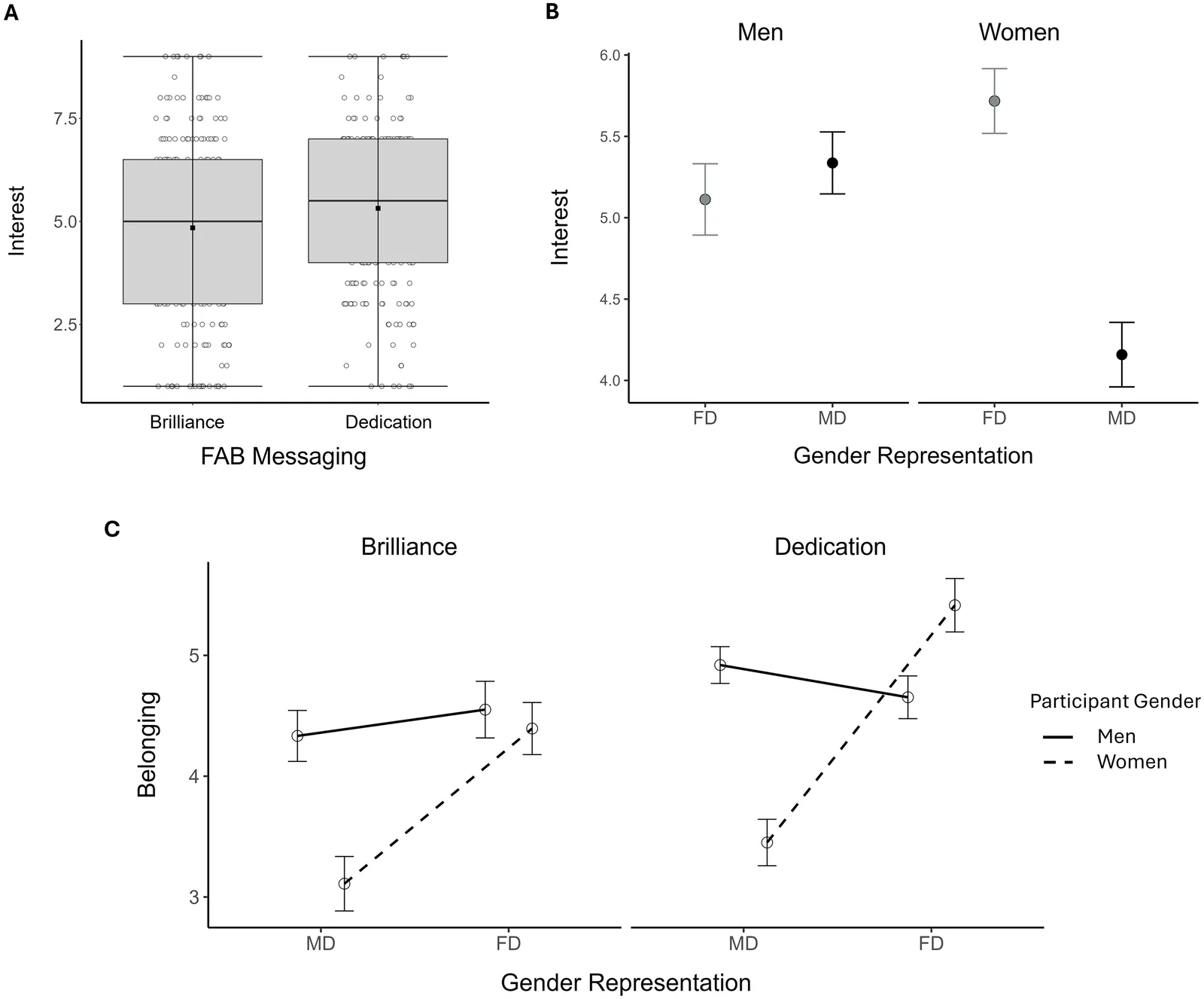
Participants’ mean ratings of interest and anticipated belonging in Study 3. *Note.* Interest ratings (average of 1-9 ratings for two items) varied as a function of FAB Messaging (A), as well as Gender Representation (Female-Dominated [FD] vs. Male-Dominated [MD]) and Participant Gender (B). Anticipated belonging (average of 1-9 ratings for two items) varied as a function of messaging, gender representation, and participant gender (C). Horizontal jitter was applied to plots (C) to enhance visual discrimination. Error bars represent SE.

A 2 (FAB messaging: brilliance vs. dedication) × 2 (gender representation: male-dominated vs. female-dominated) × 2 (participant gender: men vs. women) between-subjects ANOVA revealed significant main effects of FAB messaging, *F*(1, 391) = 6.14, *p* = .014, *η*_*p*_^*2*^ = .015, *f* = .124 (see Fig 3A), and gender representation, *F*(1, 391) = 11.27, *p* < .001, *η*_*p*_^*2*^ = .028, *f* = .170, on participants’ interest ratings. There was also a significant interaction between the gender representation condition and participant gender, *F*(1, 391) = 20.14, *p* < .001, *η*_*p*_^*2*^ = .049, *f* = .227 (see Fig 3B). No other effects reached statistical significance (see S4 File).

Participants expressed greater interest in jobs emphasizing dedication rather than brilliance, replicating the FAB effect. Importantly, however, gender representation also mattered: we found an additional effect of gender representation, which interacted with participant gender. Specifically, women were more interested in female-dominated jobs compared to male-dominated jobs (*t*[391] = 5.55, *p*_tukey_ < .001) and, when female-dominated, interest level even matched that of men’s (*ts*[391] > 2.18, all *ps* > .132). Unlike women, men reported equal interest in male- and female-dominated jobs (*t*[391] = 0.80, *p*_tukey_ = .855).

We also tested for effects of MCC and PM on participants’ interest for each job. An ANCOVA revealed a significant effect of PM, *F*(1, 388) = 250.62, *p* < .001, *η*_*p*_^*2*^ = .392, *f* = .806 (MCC was not statistically significant, *F*[1, 388] = 3.14, *p* = .077, *η*_*p*_^*2*^ = .008, *f* = .090), such that greater PM predicted greater interest in applying for the job. The effect of gender representation remained statistically significant, *F*(1, 388) = 5.44, *p* = .020, *η*_*p*_^*2*^ = .014, *f* = .119, though not the interaction between gender representation and participant gender (*F*[1, 388] = 0.20, *p* = .655, *η*_*p*_^*2*^ = .001, *f* = .032), suggesting that greater interest in female-dominated employment did not differ between men and women. FAB messaging was also not significant (*F*[1, 388] = 0.39, *p* = .534, *η*_*p*_^*2*^ = .001, *f* = .063). (See S4 File for regression analyses.) These results suggest that job interest is determined by gender representation more than FAB messaging. In general, there was greater interest in female-dominated jobs, an effect that was robust to MCC and PM.

#### How do FAB and actual gender representation affect belonging?

Parallel analyses were conducted on participants’ ratings of anticipated belonging. An ANOVA, with FAB messaging and gender representation conditions, as well as participant gender, as between-subjects factors, revealed significant main effects of each (FAB messaging, *F*[1, 391] = 12.48, *p* < .001, *η*_*p*_^*2*^ = .031, *f* = .179; gender representation, *F*[1, 391] = 30.38, *p* < .001, *η*_*p*_^*2*^ = .072, *f* = .278; participant gender, *F*[1, 391] = 12.94, *p* < .001, *η*_*p*_^*2*^ = .032, *f* = .181). There were also significant two- and three-way interactions: gender representation by participant gender, *F*[1, 391] = 32.28, *p* < .001, *η*_*p*_^*2*^ = .076, *f* = .285, and FAB messaging by gender representation by participant gender, *F*[1, 391] = 4.03, *p* = .046, *η*_*p*_^*2*^ = .010, *f* = .100 (see Fig 3C; see S4 File for item analyses and regression models).

To better understand the higher-level three-way interaction, we conducted post-hoc analyses. These analyses revealed that women reported greater anticipated belonging for jobs that were female-dominated compared to male-dominated (*t*s[391] > 3.30, *ps*_*tukey*_ < .03). Strikingly, anticipated belonging in women was even higher for a female-dominated job that emphasized brilliance compared to a male-dominated job that emphasized dedication (*t*[391] = 3.30, *p*_*tukey*_ = .023), sugges*t*ing a greater weighting of gender representation than FAB by women on future workplace appraisal. In the case of female-dominated jobs, women reported higher belonging for the job emphasizing dedication than brilliance (*t*[391] = 3.50, *p*_*tukey*_ = .012. When *t*he job was male-dominated, however, women’s anticipated belonging did not differ by FAB messaging (*t*[391] = 1.18, *p*_*tukey*_ = .937).

By contrast, men’s ratings of anticipated belonging for hypothetical jobs were largely unaffected by gender representation, or FAB messaging, for that matter. More specifically, men reported comparable levels of belonging across all conditions (*t*s[391] < 2.04, *p*s_*tukey*_ > .459); their belonging ratings were dependent neither on messaging (brilliance vs. dedication) nor representation (male- vs. female-dominated).

When accounting for potential effects of MCC and PM on anticipated belonging, the main effects of the gender representation condition (*F*[1, 388] = 18.92, *p* < .001, *η*_*p*_^*2*^ = .046, *f* = .219) and participant gender (*F*[1, 388] = 16.90, *p* < .001, *η*_*p*_^*2*^ = .042, *f* = .210) remained statistically significant, as did the two-way (gender representation by participant gender: *F*[1, 388] = 8.03, *p* = .005, *η*_*p*_^*2*^ = .020, *f* = .143) and three-way (FAB messaging by gender representation by participant gender: *F*[1, 388] = 6.16, *p* = .013, *η*_*p*_^*2*^ = .016, *f* = .128) interactions. As described above, men showed little variation in belonging ratings, with no significant posthoc effects meeting statistical significance (*p*s > .272); women’s belonging was more impacted by gender representation, with higher belonging for female-dominated than male-dominated employments, regardless of FAB messaging. There were additional effects of MCC, *F*(1, 388) = 81.39, *p* < .001, *η*_*p*_^*2*^ = .173, *f* = .458, and PM, *F*(1, 388) = 120.96, *p* < .001, *η*_*p*_^*2*^ = .238, *f* = .557, with greater MCC related to lower belonging and greater PM related to higher belonging.

Study 3 illustrates the impact of gender representation on women’s anticipated belonging, over and above that of MCC and PM. A caveat is that this effect may be more robust when participants are directly asked about belonging (e.g., “I would feel like I belong”) rather than indirectly (e.g., “I would feel like I always have to prove myself” [reverse-coded]); see S4 File). The effect of FAB messaging depended on the gender representation of the hypothetical job, though, again, this was more robust when participants were directly rather than indirectly questioned about their sense of belonging. Overall, women anticipated greater belonging in a job that emphasized dedication over brilliance, but only when the job was female-dominated. In the male-dominated case, differential FAB messaging had no effect on women’s appraisals. Importantly, however, the greatest sense of anticipated belonging among women occurred for female-dominated jobs that emphasized dedication. In total, these findings demonstrate that current gender demographics play a significant role in determining future belonging. Women clearly preferred female-dominated over male-dominated professions, regardless of FAB messaging. Nevertheless, when female-dominated, messaging had added value, such that a job emphasizing dedication resulted in increased belonging compared to one emphasizing brilliance.

## Study 4

In a final, preregistered study, we extend our previous findings in which gender representation was found to impact participants’ interest and anticipated belonging. Here we tested whether sensitivity to gender representation scales with the proportion of male to female employees by varying gender ratios parametrically. If women, in particular, use gender composition as a cue when evaluating future employment, then interest and belonging should increase as female representation increases. At the same time, it is also possible that, despite sensitivity to the male-female ratio, jobs in which the distributions are too disproportionate may be unappealing. Regardless, Study 4 provides a quantitative test of how current gender demographics may shape interest and belonging for future employment.

### Method

#### Participants.

A total of 1141 participants were recruited through Prolific. Of these, 150 were excluded from analyses because they failed the manipulation checks (*n* = 136) or did not identify as male or female (*n* = 14). The final sample comprised 991 participants (*M*_*age*_ = 39.5 years, range = 18–75 years; 496 women, 495 men). Sample size was based on a priori power analysis for an ANOVA with main effects and interactions (power = .80, α = .05), which indicated a sample size of 957 would be sufficient to detect a medium-sized effect. Eligibility criteria and compensation were identical to the previous studies.

A sensitivity analysis (G*Power 3.1) indicated that, with *N* = 991, and 24 between-subjects groups (2[FAB messaging] × 2[gender majority] × 3[gender ratio] × 2[participant gender]), the design was powered to detect a minimum effect of *f²* = 0.151. Although this reflects a large effect size by conventional standards [Cohen, 1988], it is consistent with expected reductions in power for multifactorial designs [29].

#### Procedure.

Experimental conditions—FAB messaging (brilliance vs. dedication), gender majority (male- dominated vs. female-dominated), and gender ratio (60:40 vs. 75:25 vs. 90:10)—were crossed for a total of 12 groups. Participants were randomly assigned to one of the 12 between-subjects groups. FAB messaging was identical to that of previous studies, in that participants were presented with job advertisements emphasizing the importance of either brilliance or dedication. Gender representation was manipulated in two ways: majority and ratio. That is, participants could be presented with an ad that described either majority male or female employees in one of three ratios. For example, for the 60:40 distribution, participants were told that employees were either 60% men and 40% women (male-dominated) or 60% women and 40% men (female-dominated). All other procedural aspects were identical to Study 3, including the manipulation checks.

### Results

Analyses were conducted on composite scores for the different measures, as in the previous studies. Items within measure were significantly correlated: interest (*r*[988] =.842, *p* < .001), belonging (*r*[986] =.278, *p* < .001), MCC (*rs*[979, 982] >.548, *ps* < .001) and PM (*r*[981] =.593, *p* < .001). Cronbach’s alphas were high for measures of interest (.914), MCC (.814), and PM (.744), but lower for belonging (.433). (See S5 File for belonging item analyses.)

#### Does interest in future employment depend on the ratio of the gender majority?

**Fig 4:**
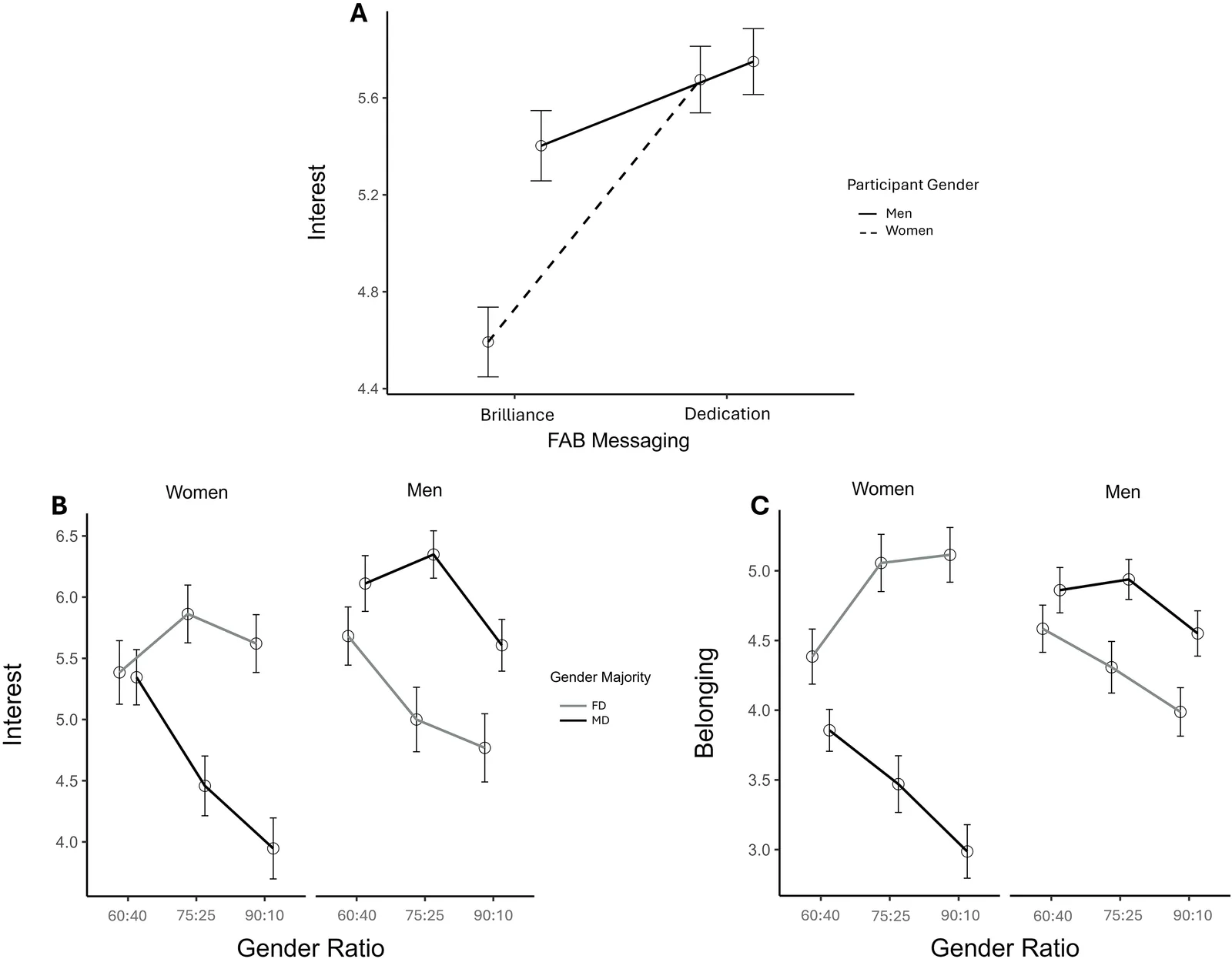
Participants’ mean ratings of interest and anticipated belonging in Study 4. *Note.* Interest and belonging ratings are average (mean) scores for items within each measure. Interest varied as a function of FAB Messaging and Participant Gender (A). Interest (B) and Anticipated Belonging (C) varied as a function of Gender Majority (Male- vs. Female-Dominated [MD and FD, respectively]), Gender Ratio, and Participant Gender. Horizontal jitter was applied to plots. Error bars represent SE.

To explore how the ratio of the gender majority was used by participants when considering future employment, we conducted an ANOVA with FAB messaging (brilliance vs. dedication), gender majority (male- vs. female-dominated), gender ratio (60:40, 75:25, vs. 90:10), and participant gender (male vs. female) as between-subjects factors on ratings of interest. This analysis revealed significant main effects of FAB messaging (*F*[1, 967] = 26.97, *p* < .001, *η*_*p*_^*2*^ = 0.027, *f* = .167), gender ratio (*F*[2, 967] = 7.64, *p* < .001, *η*_*p*_^*2*^ = 0.016, *f* = .128), and participant gender (*F*[1, 967] = 11.11, *p* < .001, *η*_*p*_^*2*^ = 0.011, *f* = .105). There were also significant two-way interactions between FAB messaging and participant gender (*F*[1, 967] = 7.26, *p* = .007, *η*_*p*_^*2*^ = 0.007, *f* = .084, see Fig 4A), and between gender majority and participant gender (*F*[1, 967] = 50.03, *p* < .001, *η*_*p*_^*2*^ = 0.049, *f* = .227). Finally, there was a significant three-way interaction among gender majority, gender ratio, and participant gender (*F*[2, 967] = 6.23, *p* = .002, *η*_*p*_^*2*^ = 0.013, *f* = .115, see Fig 4B). No other effects were statistically significant (see S5 File).

Post-hoc comparisons of the two-way interaction between FAB messaging and participant gender (Fig 4A) revealed that women, but not men, were more interested in a job opportunity that emphasized dedication rather than brilliance (*t*[967] = 5.58, *p*_*tukey*_ < .001). Indeed, women reported less interest than men in a job highlighting brilliance (*t*[967] = −4.27, *p*_*tukey*_ < .001), but there was no difference between genders when the job ad highlighted dedication (*t*[967] = −0.45, *p*_*tukey*_ = .969). Men’s interest ratings did not differ between dedication and brilliance conditions (*t*[967] = 1.77, *p*_*tukey*_ = .290).

We also unpacked the three-way interaction involving gender majority, gender ratio, and participant gender on participants’ interest ratings (see Fig 4B). Post-hoc comparisons revealed that women indicated the most interest in future employment when the employees of the advertised job were majority female compared to majority male (*t*s[967] > 4.18, *p*s_*tukey*_ < .01). The exception was when the ratio was 60:40; here, women showed comparable interest (*t*[967] =.37, *p*_*tukey*_ = 1.00). Men showed greater interest for male-dominated jobs, but this effect was only significant for the intermediate ratio, wherein 75% majority male employees elicited greater interest among men compared to 75% majority female employees (*t*[967] = 4.14, *p*_*tukey*_ = .002). Moreover, women’s interest was comparable to that of men’s (for male-dominated ratios) when the job ad indicated a majority of female employees, regardless of gender ratio (*t*s[967] < 2.72, *p*s_*tukey*_ > .217).

As in our previous studies, we also examined whether the effects of FAB messaging and gender representation on participants’ interest withstood effects of MCC and PM. An ANCOVA revealed significant effects of MCC (*F*[1, 963] = 37.89, *p* < .001, *η*_*p*_^*2*^ = 0.038, *f* = .200), and PM (*F*[1, 963] = 533.68, *p* < .001, *η*_*p*_^*2*^ = 0.357, *f* = .745) on interest ratings. Importantly, this analysis also revealed that the two-way interactions between FAB messaging and participant gender (*F*[1, 963] = 4.29, *p* = .039, *η*_*p*_^*2*^ = 0.004, *f* = .064) and between gender majority and participant gender (*F*[1, 963] = 4.08, *p* = .044, *η*_*p*_^*2*^ = 0.004, *f* = .063) remained significant. The three-way interaction between gender majority, gender ratio, and participant gender was no longer significant (*F*[2, 963] = 2.42, *p* = .090; *η*_*p*_^*2*^ = 0.005, *f* = .071). (See S5 File for regression model.) Altogether, these results demonstrate separate effects for FAB messaging and gender representation on job interest among men and women, even when accounting for MCC and PM.

#### How does ratio of the gender majority affect belonging?

Following the analyses of participants’ interest ratings, we conducted a between-subjects ANOVA to test for main effects of FAB messaging (brilliance vs. dedication), gender majority (male- vs. female-dominated), gender ratio (60:40, 75:25, vs. 90:10), participant gender (male vs. female), and their corresponding interactions, on participants’ ratings of anticipated belonging. This analysis revealed significant main effects of FAB messaging (*F*[1, 967] = 25.48, *p* < .001, *η*_*p*_^*2*^ = 0.026, *f* = .165), gender majority (*F*[1, 967] = 21.18, *p* < .001, *η*_*p*_^*2*^ = 0.021, *f* = .146), gender ratio (*F*[2, 967] = 3.25, *p* = .039, *η*_*p*_^*2*^ = 0.007, *f* = .084), and participant gender (*F*[1, 967] = 35.35, *p* < .001, *η*_*p*_^*2*^ = 0.014, *f* = .119). There were also significant two-way interactions between gender majority and gender ratio (*F*[2, 967] = 3.23, *p* = .040, *η*_*p*_^*2*^ = 0.007, *f* = .084) and between gender majority and participant gender (*F*[1, 967] = 87.96, *p* < .001, *η*_*p*_^*2*^ = 0.083, *f* = .299). Finally, as with interest ratings, there was a significant three-way interaction among gender majority, gender ratio, and participant gender (*F*[2, 967] = 6.77, *p* < .001, *η*_*p*_^*2*^ = 0.014, *f* = .119) on participants’ ratings of anticipated belonging (see Fig 4C). (See S5 File for belonging item analyses and regression models.)

To summarize, participants reported a greater sense of belonging for jobs in which dedication, rather than brilliance, was emphasized. The higher-level interaction involving gender majority, gender ratio, and participant gender (Fig 4C) revealed that women, in particular, anticipated more belonging for jobs that comprised a higher proportion of same-gender employees compared to opposite-gender employees (90:10 and 75:25, *t*s[967] > 6.32, *p*s_tukey_ < .001; 60:40, *t*[967] = 2.39, *p*_tukey_ = .416). Moreover, women’s anticipated belonging was comparable to that of men’s when their respective gender was represented by the majority of employees (*t*s[967] < 2.40, *p*s_tukey_ > .406). By contrast, women reported lower anticipated belonging than men when opposite gender employees were in the majority, an effect that scaled with the gender ratio of employees (90:10, *t*[967] = 3.83, *p*_tukey_ = .008; 75:25, *t*[967] = 3.39, *p*_tukey_ = .035; 60:40, *t*[967] = 2.98, *p*_tukey_ = .118).

Men showed less variation in anticipated belonging as a function of the gender majority and gender ratio; there were no significant rating differences for gender ratio within either female- (*t*s[967] < 2.29, *p*s_tukey_ > .481) or male-dominated (*t*s[967] < 1.30, *p*s_tukey_ > .979) conditions. Nevertheless, anticipated belonging was generally higher for male-dominated compared to female-dominated employment, though none of the pairwise comparisons per ratio reached statistical significance.

When accounting for effects of MCC and PM on participants’ anticipated belonging, an ANCOVA revealed significant effects of MCC (*F*[1, 963] = 342.83, *p* < .001, *η*_*p*_^*2*^ = 0.263, *f* = .597) and PM (*F*[1, 963] = 317.38, *p* < .001, *η*_*p*_^*2*^ = 0.248, *f* = .574). Moreover, the three-way interaction involving gender majority, gender ratio, and participant gender remained statistically significant (*F*[2, 963] = 5.03, *p* = .007, *η*_*p*_^*2*^ = 0.010, *f* = .100), though this effect was stronger for the direct measure of belonging (see S5 File). No effects related to FAB messaging were significant (*p*s > .110, *f*s < .055; S5 File).

Altogether, these results confirm the robustness of gender representation on feelings of belonging, as anticipated in future employment. Men and women expressed greater anticipated belonging for jobs with their respective gender majority, though these effects were larger in women. Women reported higher belonging ratings for female-dominated jobs, with stronger effects for more disproportionate ratios (e.g., 90:10 vs. 60:40), which were robust to MCC and PM.

## General discussion

Existing research points to the possibility that male-dominated fields would become more palatable for women if beliefs about what it takes to succeed were to be shifted—for example, by emphasizing dedication rather than brilliance since brilliance is thought to be more male-stereotypic [9–11,24,30]. The present research lends support to this possibility by demonstrating that, in some contexts, women show greater interest and a sense of belonging in occupations that place a higher premium on dedication rather than brilliance. Importantly, the present research also suggests that shifting ability beliefs alone may be insufficient for promoting female representation. Consistent with this impression, recent studies suggest that workplace appraisals related to MCC and PM are additional considerations by people [11,17], which the current findings confirm. Beyond these factors, the present investigation extends prior work by highlighting the added value of considering the demographic composition of male and female employees.

Across four studies, we found that people’s perceptions of gender demographics predicted both the real-world male-female breakdown of employees (Study 1) and their intentions to pursue employment in advertised jobs (Studies 2–4). Moreover, we found that estimated and known gender demographics explained interest and anticipated belonging over and above beliefs about the requisite qualities needed for success (i.e., brilliance vs. dedication). Strikingly, there were even conditions in which gender demographics outweighed FAB in people’s considerations of future employment. In Study 3, for example, women anticipated greater belonging in a female-dominated “brilliance job” compared to a male-dominated “dedication job.” And in Study 4, information about the gender (im)balance was a consistent predictor of both interest and anticipated belonging of future employment, whereas FAB was not. Overall, these results are consistent with homophily and relational-demography accounts [26], which posit that when individuals anticipate greater in-group representation, they may infer value congruence and reduced threat, thereby increasing interest and belonging. In this sense, gender ratios likely operate as a proxy for workplace climate—capturing expectations about support, inclusivity, and competitiveness—rather than as a purely demographic preference.

That said, we would not argue that FAB is unimportant to future representation. Replicating other research [9–11], we found that opportunities highlighting dedication were preferred over those highlighting brilliance. At the same time, our results underscore the importance of gender demographics. Why might knowledge of an occupation’s gender breakdown be so meaningful? As foreshadowed above, women may prefer employment opportunities in which they anticipate in-group membership [25–27,31]. Like other group categories, gender may signal the likelihood of shared traits and values, consistent with the importance of prototype matching (PM) in our and others’ data [11,14,20]. Beliefs that job-related success is based more on dedication than brilliance may be one such value that women share. But there are other relevant characteristics that can make jobs (un)appealing. Other research has highlighted issues such as work-life balance and services such as family support amenities, which may be particularly important for women [32–36]. The present findings are also consistent with a role for workplace culture, such as MCC, in shaping interest and belonging [16,17,19]. Anticipating an environment that is toxic and competitive—which is more likely when dominated by men—may be especially aversive for women, who are often socialized into contrasting interpersonal norms [18,27,37]. Women may treat gender as a proxy for these general concerns and, thus, place greater weight on gender demographics when considering future employment.

Men also showed homophily effects in that they expressed somewhat greater interest and belonging for environments in which male employees were in the majority (Study 4). Importantly, gender representation influenced both men and women, although the magnitude and consistency of these effects varied across studies. In Study 2, for example, expected female representation predicted interest in men. In Study 4, by contrast, responses scaled with the degree of imbalance for both genders, with women appearing more sensitive to extreme imbalances. These patterns suggest that gender demographics matter broadly, but may carry different psychological weight depending on context and degree of imbalance. Consideration of the gender demographics may reasonably impact men and women differently. One possibility for this difference may be that concerns about gender demographics are less at the forefront of men’s minds (though not necessarily absent), given their higher average status in many domains. Indeed, men often occupy leadership roles and receive greater pay even in female-dominated occupations [38,39], and work culture tends to default to male gender norms [26]. Women, by contrast, may be more sensitive to the consequences of gender representation.

Nevertheless, there are open questions about how men and women think about gender breakdowns across occupations. In Study 4, the gender imbalance varied from what might be considered a relatively small imbalance (60% vs. 40%) to a large imbalance (90% vs. 10%). Both men and women appeared tolerant of the smaller imbalance, with reported interest and anticipated belonging that did not differ dramatically depending on whether men or women were in the 60% majority. However, women were far more affected than men when the imbalance was high. Indeed, the difference between a 90% female- versus 90% male-majority had greater impact on women than men. Interest in applying for the advertised job as well as the anticipated belonging during employment were lower for women compared to men when the opposite gender (men and women, respectively) was in the majority. These findings suggest greater consequences for women than men as the gender imbalance increases in the workplace.

To be clear, we are *not* suggesting that increasing female representation is the panacea for combating gender bias in the workplace or elsewhere. Although greater female representation was associated with greater interest and anticipated belonging, FAB also played a role, which is consistent with other studies [9,11]. In at least one case (Study 3), the effects of gender demographics and FAB appeared additive, with women expressing the greatest anticipated belonging for female-dominated jobs that emphasized dedication. Even in professions where women have become better represented, men may still be viewed as more competent and deserving of higher raises compared to women [40,41]. Thus, the greatest impact on future representation is likely to come when considering the separate and combined influences of current gender demographics and FAB, which are not mutually exclusive. For diversity, equity, and inclusion (DEI) efforts, increasing gender representation may constitute one aspect of the overall mission (diversity); perhaps others (equity and inclusion) rely on other processes, such as changing people’s beliefs about the abilities required for success and workplace fit [26]. Future research would do well to consider the combined effects of DEI to build a more inviting professional landscape with long-term support for those who have been underrepresented.

By way of conclusion, we acknowledge some constraints on the generality of our findings [42]. First, we did not consider all identity factors that may be relevant when assessing occupational interest and belonging. Our analyses treated gender as a binary (men vs. women), leaving open questions about how non-binary or gender queer individuals would respond. We also did not test for differences in race/ethnicity, so it is unclear whether gender representation is equally relevant to all cultural groups. Indeed, the racial distribution of job holders may be weighted more strongly than gender for racially marginalized groups or, at the very least, intersect with gender in important ways. Following this point, an interesting area for future research would be to examine generational change. The age range of participants was quite large in the present research and although we confirmed that the reported effects held when excluding participants over 65, we did not systematically examine differences across younger and older adults. One might imagine differences in how people of different generations regard female (under)representation. We also note that many of the effects we observed, or were powered to detect, are large by conventional standards. This might be viewed as a limitation. Yet given the practical nature of our research question—how gender representation influences occupational engagement—it is reasonable to expect that only effects of a meaningful magnitude are likely to yield real-world consequences. In this respect, pursuing large, practically significant effects is not only justified but may also be necessary. Moreover, the job ads used in our studies were hypothetical. Although this procedure is common in this literature (e.g., [13]), we and others should consider testing these questions in real-world job scenarios to assess the impact of gender representation more directly. Finally, a further limitation concerns measurement of anticipated belonging. The abbreviated two-item belonging scale yielded modest internal reliability across our studies. Nevertheless, we retained the composite measure for continuity with prior work and because the items consistently patterned with theoretically predicted variables (e.g., gender representation, FAB). Importantly, all key effects replicated across studies, suggesting that the observed relations are robust despite measurement noise.

Despite these constraints, the present research identifies clear and actionable levers for fostering gender diversity in professional domains. Representation itself is a powerful signal: the visible presence of women can enhance interest and belonging, thereby perpetuating future inclusion. At the same time, shifting the narrative from innate brilliance to dedication offers another pathway toward equity. Together, these findings suggest that demographic and cultural change are mutually reinforcing—achieving one without the other may be insufficient. Jointly, however, they can cultivate workplaces in which all individuals, regardless of gender, see themselves as capable of thriving.

## Supporting information

S1 File SI-1 Measures. (DOCX)

S2 File SI-2 Study 1 analyses. (DOCX)

S3 File SI-3 Study 2 analyses. (DOCX)

S4 File SI-4 Study 3 analyses. (DOCX)

S5 File SI-5 Study 4 analyses. (DOCX)
