# Supplementary material for: Gender representation begets gender representation
Source: PLoS One. 2026 Aug 26;21(8):e0351865. doi: 10.1371/journal.pone.0351865 (PMC13514026; doi:10.1371/journal.pone.0351865)
Supplement: Supplementary file 1 [file pone.0351865.s001.docx]

SI – 1: Measures

Participants were given the following measures in each study.

|  | **Study 1** | **Study 2** | **Study 3** | **Study 4** |
| --- | --- | --- | --- | --- |
| Estimate of gender distribution | ✓ | ✓ |  |  |
| Field-specific ability beliefs (FAB) | ✓ |  |  |  |
| Belonging | ✓ | ✓ | ✓ | ✓ |
| Prototype matching | ✓ | ✓ | ✓ | ✓ |
| Masculinity contest culture | ✓ | ✓ | ✓ | ✓ |
| Interest |  | ✓ | ✓ | ✓ |

Instructions and individual items for each measure are presented below.

Estimate of gender distribution

Please indicate, using the sliding scale, your estimate of the gender distribution within the occupation listed above, where the amount of blue indicates percentage of women and amount of white indicates percentage of men.

Field-specific ability beliefs (FAB)

1 = “strongly disagree” to 7 = “strongly agree”

Please rate the extent to which you agree with the following statements, considering the occupation listed above.

- Being a [ ] requires a special aptitude that just can’t be taught.
- If you want to succeed as a [ ], hard work alone just won’t cut it; you need to have an innate gift or talent.
- With the right amount of effort and dedication, anyone can become a successful [ ]. (R)^[1]^
- When it comes to being a [ ], the most important factors for success are motivation and sustained effort; raw ability is secondary. (R)

Belonging^[2]^

*Study 1*

- With the appropriate training and resources, I feel like I would belong in this occupation.
- With the appropriate training and resources, I would feel like I always have to prove myself in this occupation. (R)

*Studies 2-4*

Imagine that you are [hired for this job]. How would you feel?

- I would feel like I belong.
- I would feel like I always have to prove myself. (R)

Prototype matching (PM)^[3]^

*Studies 1 – 4*

- How similar do you think you are to the other people who hold this occupation?

*Studies 2 - 4*

- How well do you think you match the requirements for this job?

Masculinity contest culture (MCC) ^[4]^

- In this job, admitting you don’t know the answer looks weak. (“Show no weakness.”)
- In this job, taking days off is frowned upon. (“Put work first.”)
- In this job, one person’s loss is another person’s gain. (“Dog eat dog.”)

Interest

1 = “not at all interested” to 9 = “extremely interested”

- Assuming you were looking for a [job], how likely would you be to apply for this particular [job]?
- Assuming you were looking for a long-term career, how likely would you be to consider turning this [job] into such a career?

**Job advertisements.** Two job advertisements were used in Studies 2-4. In both cases, participants were given the following instructions:

Please imagine that you have been out of a job for a couple of weeks and are looking for a new job. The advertisement that follows is from a popular job website. Please read the ad carefully. You will then be asked to evaluate this job opportunity.

Brilliance condition

We are looking for a number of applicants to fill positions in our workforce. Because of the work we do, we are looking for a candidate who has a high IQ, superior reasoning skills, and a knack for big, bold ideas. That is, we’d like to hire someone whose intellectual abilities stand out from those of their peers. Our work environment values and emphasizes employees’ natural intelligence, expecting everyone to perform at their peak. Therefore, we are especially interested in candidates who demonstrate an inherent aptitude for this position.

Dedication condition

We are looking for a number of applicants to fill positions in our workforce. Because of the work we do, we are looking for a motivated candidate with a strong work ethic and a commitment to completing their work well. That is, we’d like to hire someone who has demonstrated sustained dedication in their past positions. Our work environment values and emphasizes employees’ consistent effort to achieve goals, expecting everyone to continuously improve their work performance. Therefore, we are especially interested in candidates who demonstrate dedication for their work.

*Study 3*

In Study 3, demographic information was presented at the end of each job ad. For both conditions (i.e., male-dominated vs. female-dominated), three out of four facts were identical. These were:

- Our employees’ average retirement age is 65 years old.
- Our employees report that their typical work week is Monday through Friday, though some weekend hours may be required.
- All employees receive health benefits, including access to medical, dental, and vision insurance.

The last fact is what differed between the two conditions. In the male-dominated condition, participants were presented with the statement: 75% of our employees are men and 25% are women. In the female-dominated condition, participants were presented with the statement: 75% of our employees are women and 25% are men.

*Study 4*

The setup for Study 4 was identical to that of Study 3 except for differences in the stated gender demographics of employees. That is, in the male-dominated conditions, participants were presented with the statement: [ ]% of our employees are men and [ ]% are women. In the female-dominated conditions, they were presented with the statement: [ ]% of our employees are women and [ ]% are men. The gender ratios used were 90:10, 75:25, and 60:40.

1. (R) indicates items were reverse coded.
2. In Study 1, belonging was rated on a 1 = “strongly disagree” to 7 = “strongly agree” scale to be consistent with the FAB measure. It was rated on a 1 to 9 scale in Studies 2-4 to be consistent with all other measures.
3. In Study 1, PM was rated on a 1 = “not at all similar” to 7 = “very similar” scale to be consistent with the FAB measure. It was rated on a 1 to 9 scale in Studies 2-4 to be consistent with all other measures.
4. In Study 1, MCC was rated on a 1 = “strongly disagree” to 5 = “strongly agree” scale (as in Glick et al., 2018). It was rated on a 1 to 9 scale in Studies 2-4 to be consistent with all other measures.
