# Supplementary material for: Gender representation begets gender representation
Source: PLoS One. 2026 Aug 26;21(8):e0351865. doi: 10.1371/journal.pone.0351865 (PMC13514026; doi:10.1371/journal.pone.0351865)
Supplement: Supplementary file 2 [file pone.0351865.s002.docx]

SI – 2: Study 1 Analyses

**Table S1**

Study 1 regression model predicting female participants’ anticipated belonging

|  | ***R^2^* = .351**, ***f^2^*  = .459** | | | |
| --- | --- | --- | --- | --- |
| **Predictor** | ***β*** |  | **t** | **p** |
| Representation Estimate | 0.147 |  | 7.35 | **<.001** |
| FAB | -0.205 |  | -10.72 | **<.001** |
| MCC | -0.217 |  | -11.17 | **<.001** |
| PM | 0.467 |  | 23.23 | **<.001** |
| *Note.* Standardized *β* coefficients are reported for all variables. | | | | |

**Table S2**

Study 1 regression model predicting male participants’ anticipated belonging

|  | ***R^2^* = .213, *f*^2^ = .271** | | |
| --- | --- | --- | --- |
| **Predictor** | ***β*** | **t** | **p** |
| Representation Estimate | 0.020 | 0.72 | 0.475 |
| FAB | -0.259 | -9.41 | **<.001** |
| MCC | -0.235 | -8.30 | **<.001** |
| PM | 0.355 | 12.64 | **<.001** |
| Note. Standardized *β* coefficients are reported for all variables. | | | |

**Additional Analyses of Belonging**

Items within FAB (*r*s[2832] > .346, *p*s < .001) and MCC (*r*s[2832] > .325, *p*s < .001) measures were significantly correlated. These measures also had acceptable internal reliability, as indicated by Cronbach’s alphas FAB (0.834) and MCC (0.648). The two items in the Belonging measure, however, were not significantly correlated with one another, and Cronbach’s alpha was poor. We thus conducted additional analyses for each Belonging item.

Trial-level multiple regression analyses were largely consistent with those for the composite measure of Belonging, though only the second item (“With the appropriate training and resources, I would feel like I always have to prove myself in this occupation” [reverse coded]; *β* = 0.130, *t* = 7.38, *p* < .001), not the first item (“With the appropriate training and resources, I feel like I would belong in this occupation”; *β* = 0.023, *t* = 1.64, *p* = .102), was significantly predicted by participants’ estimates of gender representation.

The interactions between FAB and participant gender were significant when considered separately by each Belonging item. With each item, there were significant interactions (*β* > -0.084, *t* > -2.35, *p*s < .02). For Item 1, men reported greater anticipated belonging compared to women when FAB ratings were low (both men and women reported less belonging when FAB was high). For Item 2, men reported greater anticipated belonging than women when FAB was high (both men and women reported greater belonging when FAB was low).
