# Supplementary material for: Gender representation begets gender representation
Source: PLoS One. 2026 Aug 26;21(8):e0351865. doi: 10.1371/journal.pone.0351865 (PMC13514026; doi:10.1371/journal.pone.0351865)
Supplement: Supplementary file 3 [file pone.0351865.s003.docx]

**SI – 3: Study 2 Analyses**

**Table S3**

Study 2 regression models predicting female participants’ interest in the advertised jobs

| **Predictor** | **Model 1**  **(*R^2^* = .100**, ***f^2^*  = .111)** | | | **Model 2**  **(*R^2^* = .132**, ***f^2^*  = .152)** | | | **Model 3**  **(*R^2^* = .602**, ***f^2^*  = 1.513)** | | |
| --- | --- | --- | --- | --- | --- | --- | --- | --- | --- |
|  | ***β*** | ***t*** | ***p*** | ***β*** | ***t*** | ***p*** | ***β*** | ***t*** | ***p*** |
| FAB Messaging (Dedication [1 = dedication]) | 0.630 | 3.22 | .**002** | 0.583 | 2.99 | **.004** | 0.151 | 1.071 | .287 |
| Representation Estimates |  |  |  | 0.180 | 1.84 | .069 | -0.029 | -0.412 | .681 |
| MCC |  |  |  |  |  |  | -0.221 | -3.044 | **.003** |
| PM |  |  |  |  |  |  | 0.660 | 9.046 | **<.001** |
| *Note.* Standardized *β* coefficients are reported for all variables. Reference group is Brilliance (FAB Messaging).  Predicted values differ slightly between Table S3 and Table 1 because coefficents are standardized. This approach yields highly similar but non-identical estimates. | | | | | | | | | |

**Table S4**

Study 2 regression models predicting male participants’ interest in the advertised jobs

| **Predictor** | **Model 1**  **(*R^2^* < .001**, ***f^2^*  = .001)** | | | **Model 2**  **(*R^2^* = .084**, ***f^2^*  = .092)** | | | **Model 3**  **(*R^2^* = .524**, ***f^2^*  = 1.101)** | | |
| --- | --- | --- | --- | --- | --- | --- | --- | --- | --- |
|  | ***β*** | ***t*** | ***p*** | ***β*** | ***t*** | ***p*** | ***β*** | ***t*** | ***p*** |
| FAB Messaging (Dedication [1 = dedication]) | 0.035 | 0.171 | 0.865 | 0.066 | 0.338 | .736 | -0.200 | -1.37 | .175 |
| Representation Estimates |  |  |  | 0.290 | 2.96 | **.004** | 0.165 | 2.21 | **.030** |
| MCC |  |  |  |  |  |  | -0.217 | -2.68 | **.009** |
| PM |  |  |  |  |  |  | 0.574 | 7.43 | **<.001** |
| *Note.* Standardized *β* coefficients are reported for all variables. Reference group is Brilliance (FAB Messaging). | | | | | | | | | |

**Table S5**

Study 2 regression models predicting participants’ anticipated belonging (composite measure) in the advertised jobs

| **Predictor** | **Model 1 (*R^2^* = .031, *f^2^*  = .032)** | | | **Model 2 (*R^2^* = .099, *f^2^*  = .110)** | | | **Model 3 (*R^2^* = .494, *f^2^*  = .976)** | | |
| --- | --- | --- | --- | --- | --- | --- | --- | --- | --- |
|  | ***β*** | ***t*** | ***P*** | ***β*** | ***t*** | ***P*** | ***β*** | ***t*** | ***P*** |
| FAB Messaging (Dedication [1 = dedication]) | 0.167 | 0.833 | .406 | 0.189 | 0.971 | .333 | -0.112 | -0.750 | .454 |
| Participant Gender (Female [1 = female]) | -0.261 | -1.318 | .189 | -0.213 | -1.552 | .122 | -0.144 | -1.227 | .221 |
| FAB Messaging × Participant Gender | 0.281 | 0.984 | .327 | 0.177 | 0.638 | .524 | 0.078 | 0.370 | .712 |
| Representation Estimates |  |  |  | 0.186 | 2.079 | **.039** | 0.036 | 0.520 | .604 |
| Representation Estimates × Participant Gender |  |  |  | 0.162 | 1.137 | .257 | 0.091 | 0.841 | .402 |
| MCC |  |  |  |  |  |  | -0.400 | -6.898 | **< .001** |
| PM |  |  |  |  |  |  | 0.430 | 7.581 | **< .001** |
| ***Note.*** Standardized *β* coefficients are reported for all variables. Reference groups are Brilliance (FAB Messaging) and Male (Participant Gender). | | | | | | | | | |

**Table S6**

Study 2 regression models predicting female participants’ (composite) belonging in the advertised jobs

| **Predictor** | **Model 1**  **(*R^2^* = .047**, ***f^2^*  = .887)** | | | **Model 2**  **(*R^2^* = .137**, ***f^2^*  = .159)** | | | **Model 3**  **(*R^2^* = .509**, ***f^2^*  = 1.037)** | | |
| --- | --- | --- | --- | --- | --- | --- | --- | --- | --- |
|  | ***β*** | ***t*** | ***p*** | ***β*** | ***t*** | ***p*** | ***β*** | ***t*** | ***p*** |
| FAB Messaging (Dedication [1 = dedication]) | 0.432 | 2.15 | .**035** | 0.353 | 1.82 | .072 | -0.049 | -0.313 | .755 |
| Representation Estimates |  |  |  | 0.303 | 3.10 | **.003** | 0.104 | 1.331 | .186 |
| MCC |  |  |  |  |  |  | -0.315 | -3.903 | **<.001** |
| PM |  |  |  |  |  |  | 0.515 | 6.355 | **<.001** |
| *Note.* Standardized *β* coefficients are reported for all variables. Reference group is Brilliance (FAB Messaging). | | | | | | | | | |

**Table S7**

Study 2 regression models predicting male participants’ (composite) belonging in the advertised jobs

| **Predictor** | **Model 1**  **(*R^2^* = .007**, ***f^2^*  = .007)** | | | **Model 2**  **(*R^2^* = .051**, ***f^2^*  = .054)** | | | **Model 3**  **(*R^2^* = .496**, ***f^2^*  = .984)** | | |
| --- | --- | --- | --- | --- | --- | --- | --- | --- | --- |
|  | ***β*** | ***t*** | ***p*** | ***β*** | ***t*** | ***p*** | ***β*** | ***t*** | ***p*** |
| FAB Messaging (Dedication [1 = dedication]) | 0.173 | 0.854 | 0.395 | 0.195 | 0.981 | .329 | -0.120 | -0.798 | .427 |
| Representation Estimates |  |  |  | 0.209 | 2.102 | **.038** | 0.026 | 0.345 | .731 |
| MCC |  |  |  |  |  |  | -0.507 | -6.091 | **<.001** |
| PM |  |  |  |  |  |  | 0.336 | 4.235 | **<.001** |
| *Note.* Standardized *β* coefficients are reported for all variables. Reference group is Brilliance (FAB Messaging). | | | | | | | | | |

**Table S8**

Study 2 regression models predicting participants’ anticipated belonging using Item 1 of the Belonging measure

| **Predictor** | **Model 1 (*R^2^* = .023, *f^2^*  = .024)** | | | **Model 2 (*R^2^* = .085, *f^2^*  = .093)** | | | **Model 3 (*R^2^* = .486, *f^2^*  = .946)** | | |
| --- | --- | --- | --- | --- | --- | --- | --- | --- | --- |
|  | ***β*** | ***t*** | ***P*** | ***β*** | ***t*** | ***P*** | ***β*** | ***t*** | ***P*** |
| FAB Messaging (Dedication [1 = dedication]) | 0.256 | 1.275 | .204 | 0.279 | 1.425 | .156 | 0.022 | 0.149 | .881 |
| Participant Gender [Female [1 = female]) | -0.023 | -0.116 | .908 | 0.022 | -0.746 | .457 | 0.113 | 0.116 | .908 |
| FAB Messaging × Participant Gender | 0.082 | 0.284 | .776 | -0.016 | -0.058 | .954 | -0.142 | -0.673 | .502 |
| Representation Estimates |  |  |  | 0.192 | 2.139 | **.034** | 0.085 | 1.221 | .224 |
| Representation Estimates × Participant Gender |  |  |  | 0.128 | 0.895 | .372 | 0.031 | 0.286 | .776 |
| MCC |  |  |  |  |  |  | -0.184 | -3.140 | **.002** |
| PM |  |  |  |  |  |  | 0.589 | 10.307 | **< .001** |
| ***Note.*** Standardized *β* coefficients are reported for all variables. Reference groups are Brilliance (FAB Messaging) and Male (Participant Gender). | | | | | | | | | |

**Table S9**

Study 2 regression models predicting participants’ anticipated belonging using Item 2 of the Belonging measure

| **Predictor** | **Model 1 (*R^2^* = .028, *f^2^*  = .029)** | | | **Model 2 (*R^2^* = .054, *f^2^*  = .057)** | | | **Model 3 (*R^2^* = .261, *f^2^*  = .353)** | | |
| --- | --- | --- | --- | --- | --- | --- | --- | --- | --- |
|  | ***β*** | ***t*** | ***P*** | ***β*** | ***t*** | ***P*** | ***β*** | ***t*** | ***P*** |
| FAB Messaging (Dedication [1 = dedication]) | 0.002 | 0.009 | .993 | 0.014 | 0.069 | .945 | -0.204 | -1.136 | .257 |
| Participant Gender [Female [1 = female]) | -0.399 | -2.012 | **.046** | -0.369 | -1.689 | .093 | -0.351 | -1.748 | .082 |
| FAB Messaging × Participant Gender | 0.370 | 1.294 | .197 | 0.305 | 1.070 | .286 | 0.275 | 1.084 | .280 |
| Representation Estimates |  |  |  | 0.099 | 1.087 | .278 | -0.031 | -0.368 | .713 |
| Representation Estimates × Participant Gender |  |  |  | 0.128 | 0.878 | .381 | 0.114 | 0.879 | .381 |
| MCC |  |  |  |  |  |  | -0.456 | -6.512 | **< .001** |
| PM |  |  |  |  |  |  | 0.080 | 1.169 | .244 |
| ***Note.*** Standardized *β* coefficients are reported for all variables. Reference groups are Brilliance (FAB Messaging) and Male (Participant Gender). | | | | | | | | | |
