# Supplementary material for: Gender representation begets gender representation
Source: PLoS One. 2026 Aug 26;21(8):e0351865. doi: 10.1371/journal.pone.0351865 (PMC13514026; doi:10.1371/journal.pone.0351865)
Supplement: Supplementary file 4 [file pone.0351865.s004.docx]

**SI – 4: Study 3 Analyses**

**Table S10**

Study 3 ANOVA table predicting participants’ interest in the advertised jobs

| **Measure** | ***MS*** | ***F* (1, 391)** | ***p*** | ***η²p f*** |
| --- | --- | --- | --- | --- |
| FAB Messaging | 24.71 | 6.139 | **.014** | 0.015 .124 |
| Gender Representation | 45.37 | 11.274 | **< .001** | 0.028 .170 |
| Participant Gender | 7.79 | 1.936 | .165 | 0.005 .071 |
| FAB Messaging × Gender Representation | 3.57 | 0.886 | .347 | 0.002 .045 |
| FAB Messaging × Participant Gender | 1.20 | 0.299 | .585 | 0.001 .032 |
| Gender Representation × Participant Gender | 81.03 | 20.135 | **< .001** | 0.049 .227 |
| FAB Messaging × Gender Representation × Participant Gender | 1.06 | 0.263 | .609 | 0.001 .032 |

**Table S11**

Study 3 regression table predicting participants’ interest in the advertised jobs

| **Predictor** | ***R^2^* = .094**, ***f^2^* = .104** | | |
| --- | --- | --- | --- |
|  | ***β*** | ***t*** | ***p*** |
| FAB Messaging (Dedication [1 = dedication]) | 0.250 | 1.306 | .192 |
| Gender Representation (Female Dominated [1 = female dominated]) | -0.150 | -0.780 | .436 |
| Participant Gender [Female [1 = female]) | -0.464 | -2.426 | **.016** |
| FAB Messaging × Gender Representation | 0.083 | 0.303 | .762 |
| FAB Messaging × Participant Gender | -0.204 | -0.754 | .451 |
| Gender Representation × Participant Gender | 0.765 | 2.829 | **.005** |
| FAB Messaging × Gender Representation × Participant Gender | 0.197 | 0.513 | .609 |
| *Note.* Standardized *β* coefficients are reported for all variables. Reference groups are Brilliance (FAB Messaging), Male Dominated (Gender Representation), and Male (Participant Gender). | | | |

**Table S12**

Study 3 regression table, including MCC and PM, predicting participants’ interest in the advertised jobs

| **Predictor** | ***R^2^* = .492**, ***f^2^* = .969** | | |
| --- | --- | --- | --- |
|  | ***β*** | ***t*** | ***p*** |
| FAB Messaging (Dedication [1 = dedication]) | -0.048 | -0.332 | .740 |
| Gender Representation (Female Dominated [1 = female dominated]) | 0.135 | 0.925 | .356 |
| Participant Gender [Female [1 = female]) | -0.085 | -0.587 | .557 |
| MCC | -0.072 | -1.773 | .077 |
| PM | 0.651 | 15.831 | **< .001** |
| FAB Messaging × Gender Representation | 0.005 | 0.026 | .979 |
| FAB Messaging × Participant Gender | -0.048 | -0.235 | .815 |
| Gender Representation × Participant Gender | 0.023 | 0.110 | .913 |
| FAB Messaging × Gender Representation × Participant Gender | 0.090 | 0.311 | .756 |
| *Note.* Standardized *β* coefficients are reported for all variables. Reference groups are Brilliance (FAB Messaging), Male Dominated (Gender Representation), and Male (Participant Gender). | | | |

**Table S13**

Study 3 regression table predicting participants’ belonging (composite score) in the advertised jobs

| **Predictor** | ***R^2^* = .191**, ***f^2^* = .236** | | |
| --- | --- | --- | --- |
|  | ***β*** | ***t*** | ***p*** |
| FAB Messaging (Dedication [1 = dedication]) | 0.367 | 2.035 | **.043** |
| Gender Representation (Female Dominated [1 = female dominated]) | 0.136 | 0.751 | .453 |
| Participant Gender [Female [1 = female]) | -0.776 | -4.244 | **< .001** |
| FAB Messaging × Gender Representation | -0.304 | -1.180 | .239 |
| FAB Messaging × Participant Gender | -0.154 | -0.603 | .547 |
| Gender Representation × Participant Gender | 0.668 | 2.616 | **.009** |
| FAB Messaging × Gender Representation × Participant Gender | 0.729 | 2.006 | .**046** |
| *Note.* Standardized *β* coefficients are reported for all variables. Reference groups are Brilliance (FAB Messaging), Male Dominated (Gender Representation), and Male (Participant Gender). | | | |

**Table S14**

Study 3 regression table, including MCC and PM, predicting participants’ belonging (composite score) in the advertised jobs

| **Predictor** | ***R^2^* = .544**, ***f^2^* = 1.193** | | |
| --- | --- | --- | --- |
|  | ***β*** | ***t*** | ***p*** |
| FAB Messaging (Dedication [1 = dedication]) | 0.064 | 0.462 | .644 |
| Gender Representation (Female Dominated [1 = female dominated]) | 0.295 | 2.139 | **.033** |
| Participant Gender [Female [1 = female]) | -0.426 | -3.098 | **.002** |
| MCC | -0.346 | -9.022 | **< .001** |
| PM | 0.429 | 10.998 | **< .001** |
| FAB Messaging × Gender Representation | -0.392 | -2.016 | **.044** |
| FAB Messaging × Participant Gender | -0.118 | -0.613 | .540 |
| Gender Representation × Participant Gender | 0.067 | 0.339 | .735 |
| FAB Messaging × Gender Representation × Participant Gender | 0.680 | 2.482 | **.013** |
| *Note.* Standardized *β* coefficients are reported for all variables. Reference groups are Brilliance (FAB Messaging), Male Dominated (Gender Representation), and Male (Participant Gender). | | | |

**Table S15**

Study 3 ANOVA table predicting participants’ anticipated belonging using Item 1 of the Belonging measure

| **Measure** | ***df*** | ***M^2^*** | ***F*** | ***p*** | ***η²p f*** |
| --- | --- | --- | --- | --- | --- |
| FAB Messaging | 1 | 9.209 | 2.587 | .109 | .007 .084 |
| Gender Representation | 1 | 112.291 | 31.541 | **< .001** | .075 .285 |
| Participant Gender | 1 | 9.704 | 2.726 | .100 | .007 .084 |
| FAB Messaging × Gender Representation | 1 | 0.090 | 0.025 | .874 | .000 .000 |
| FAB Messaging × Participant Gender | 1 | 4.259 | 1.196 | .275 | .003 .055 |
| Gender Representation × Participant Gender | 1 | 182.729 | 51.327 | **< .001** | .116 .362 |
| FAB Messaging × Gender Representation × Participant Gender | 1 | 9.967 | 2.799 | .095 | .007 .084 |

**Table S16**

Study 3 ANOVA table predicting participants’ anticipated belonging using Item 2 of the Belonging measure

| **Measure** | ***df*** | ***M^2^*** | ***F*** | ***p*** | ***η²p f*** |
| --- | --- | --- | --- | --- | --- |
| FAB Messaging | 1 | 51.839 | 13.289 | **< .001** | .033 .185 |
| Gender Representation | 1 | 28.846 | 7.395 | **.007** | .019 .139 |
| Participant Gender | 1 | 53.348 | 13.676 | **< .001** | .034 .188 |
| FAB Messaging × Gender Representation | 1 | 0.454 | 0.116 | .733 | .000 .000 |
| FAB Messaging × Participant Gender | 1 | 29.405 | 7.538 | **.006** | .019 .139 |
| Gender Representation × Participant Gender | 1 | 8.653 | 2.218 | .137 | .006 .078 |
| FAB Messaging × Gender Representation × Participant Gender | 1 | 7.048 | 1.807 | .180 | .005 .071 |

**Table S17**

Study 3 ANCOVA table predicting participants’ anticipanted belonging using Item 1 of the Belonging measure

| **Measure** | ***df*** | ***M^2^*** | ***F*** | ***p*** | ***η²p f*** |
| --- | --- | --- | --- | --- | --- |
| FAB Messaging | 1 | 3.760 | 1.867 | .173 | .005 .071 |
| Gender Representation | 1 | 51.380 | 25.526 | **< .001** | .062 .257 |
| Participant Gender | 1 | 5.630 | 2.795 | .095 | .007 0.84 |
| MCC | 1 | 5.040 | 2.505 | .114 | .006 .078 |
| PM | 1 | 487.770 | 242.347 | **< .001** | .384 .789 |
| FAB Messaging x Gender Representation | 1 | 1.850 | 0.918 | .339 | .002 .045 |
| FAB Messaging × Participant Gender | 1 | 2.110 | 1.050 | .306 | .003 .055 |
| Gender Representation × Participant Gender | 1 | 35.240 | 17.508 | **< .001** | .043 .212 |
| FAB Messaging × Gender Representation × Participant Gender | 1 | 9.170 | 4.556 | **.033** | .012 .110 |

**Table S18**

Study 3 ANCOVA table predicting participants’ anticipated belonging using Item 2 of the Belonging measure

| **Measure** | ***df*** | ***M^2^*** | ***F*** | ***p*** | ***η²p f*** |
| --- | --- | --- | --- | --- | --- |
| FAB Messaging | 1 | 1.659 | 0.569 | .451 | .001 .032 |
| Gender Representation | 1 | 5.431 | 1.863 | .173 | .005 .071 |
| Participant Gender | 1 | 43.643 | 14.972 | **< .001** | .037 .196 |
| MCC | 1 | 304.723 | 104.539 | **< .001** | .212 .519 |
| PM | 1 | 3.734 | 1.281 | .258 | .003 .055 |
| FAB Messaging × Gender Representation | 1 | 0.289 | 0.099 | .753 | .000 .000 |
| FAB Messaging × Participant Gender | 1 | 24.789 | 8.504 | **.004** | .021 .146 |
| Gender Representation× Participant Gender | 1 | 0.063 | 0.022 | .883 | .000 .000 |
| FAB Messaging × Gender Representation× Participant Gender | 1 | 5.719 | 1.962 | .162 | .005 .071 |
