# Supplementary material for: Gender representation begets gender representation
Source: PLoS One. 2026 Aug 26;21(8):e0351865. doi: 10.1371/journal.pone.0351865 (PMC13514026; doi:10.1371/journal.pone.0351865)
Supplement: Supplementary file 5 [file pone.0351865.s005.docx]

**SI – 5: Study 4 Analyses**

**Table S19**

Study 4 ANOVA table predicting participants’ interest in the advertised jobs

| **Measure** | ***df*** | ***M^2^*** | ***F*** | ***p*** | ***η²p f*** |
| --- | --- | --- | --- | --- | --- |
| FAB Messaging | 1 | 123.757 | 26.968 | **< .001** | 0.027 .167 |
| Gender Majority | 1 | 2.567 | 0.559 | .455 | 0.001 .032 |
| Gender Ratio | 2 | 35.041 | 7.636 | **< .001** | 0.016 .126 |
| Participant Gender | 1 | 50.974 | 11.108 | **< .001** | 0.011 .107 |
| FAB Messaging × Gender Majority | 1 | 5.179 | 1.129 | .288 | 0.001 .032 |
| FAB Messaging × Gender Ratio | 2 | 1.151 | 0.251 | .778 | 0.001 .032 |
| Gender Majority × Gender Ratio | 2 | 7.710 | 1.680 | .187 | 0.003 .055 |
| FAB Messaging × Participant Gender | 1 | 33.301 | 7.257 | **.007** | 0.007 .084 |
| Gender Majority × Participant Gender | 1 | 229.589 | 50.030 | **< .001** | 0.049 .228 |
| Gender Ratio × Participant Gender | 2 | 0.511 | 0.111 | .895 | 0.000 .015 |
| FAB Messaging × Gender Majority × Gender Ratio | 2 | 3.651 | 0.796 | .452 | 0.002 .045 |
| FAB Messaging × Participant Gender × Gender Ratio | 2 | 0.584 | 0.127 | .880 | 0.000 .016 |
| FAB Messaging × Gender Majority × Participant Gender | 1 | 1.483 | 0.323 | .570 | 0.000 .018 |
| Gender Majority × Gender Ratio × Participant Gender | 2 | 28.587 | 6.229 | **.002** | 0.013 .114 |
| FAB Messaging × Gender Majority × Gender Ratio × Participant Gender | 2 | 5.769 | 1.257 | .285 | 0.003 .055 |

**Table S20**

Study 4 regression coefficients for the model predicting participants’ interest in the advertised jobs

| **Predictor** | ***β*** | ***t*** | ***p*** |
| --- | --- | --- | --- |
| **FAB Messaging** |  |  |  |
| Dedication (D) – Brilliance (B) | -.093 | -0.42 | .677 |
| **Gender Majority** |  |  |  |
| FemaleDominated (FD) – MaleDominated (MD) | -0.419 | -1.90 | .058 |
| **Gender Ratio** |  |  |  |
| 75:25 – 60:40 | -0.036 | 0.17 | .867 |
| 90:10 – 60:40 | -0.487 | -2.28 | **.023** |
| **Participant Gender** |  |  |  |
| Female – Male | -0.613 | -2.95 | **.003** |
| **FAB messaging × Gender Majority** |  |  |  |
| (D – B) × (FD – MD) | 0.441 | 1.42 | .157 |
| **FAB messaging × Gender Ratio** |  |  |  |
| (D – B) × (75:25 – 60:40) | 0.143 | 0.47 | .636 |
| (D – B) × (90:10 – 60:40) | 0.501 | 1.66 | .097 |
| **Gender Majority × Gender Ratio** |  |  |  |
| (FD – MD) × (75:25 – 60:40) | -0.238 | -0.79 | .428 |
| (FD – MD) × (90:10 – 60:40) | 0.257 | 0.84 | .401 |
| **FAB messaging × Participant Gender** |  |  |  |
| (D – B) × (Female – Male) | 0.531 | 1.80 | .073 |
| **Gender Majority × Participant Gender** |  |  |  |
| (FD – MD) × (Female – Male) | 0.370 | 1.24 | .217 |
| **Gender Ratio × Participant Gender** |  |  |  |
| (75:25 – 60:40) × (Female – Male) | -0.365 | -1.25 | .211 |
| (90:10 – 60:40) × (Female – Male) | -0.071 | -.024 | .810 |
| **FAB Messaging × Gender Majority × Gender Ratio** |  |  |  |
| (D – B) × (FD – MD) × (75:25 – 60:40) | -0.315 | -0.74 | .457 |
| (D – B) × (FD – MD) × (90:10 – 60:40) | -0.828 | -1.94 | .052 |
| **FAB Messaging × Gender Ratio × Participant Gender** |  |  |  |
| (D – B) × (75:25 – 60:40) × (Female – Male) | -0.234 | -0.57 | .572 |
| (D – B) × (90:10 – 60:40) × (Female – Male) | -0.586 | -1.40 | .163 |
| **FAB Messaging × Gender Majority × Participant Gender** |  |  |  |
| (D – B) × (FD – MD) × (Female – Male) | -0.235 | -0.55 | .581 |
| **Gender Majority × Gender Ratio × Participant Gender** |  |  |  |
| (FD – MD) × (75:25 – 60:40) × (Female – Male) | 0.856 | 2.05 | **.040** |
| (FD – MD) × (90:10 – 60:40) × (Female – Male) | 0.397 | 0.94 | .347 |
| **FAB Messaging × Gender Majority × Gender Ratio × Participant Gender** |  |  |  |
| (D – B) × (FD – MD) × (75:25 – 60:40) × (Female – Male) | 0.214 | 0.36 | .718 |
| (D – B) × (FD – MD) × (90:10 – 60:40) × (Female – Male) | 0.904 | 1.51 | .131 |

*Note.* *β* = standardized coefficient. Reference groups are Brilliance (FAB Messaging), Male Dominated (Gender Majority), 60:40 (Gender Ratio), and Male (Participant Gender).

**Table S21**

Study 4 regression coefficients, including PM and MCC, for the model predicting participants’ interest in the advertised jobs

| **Predictor** | ***β*** | ***t*** | ***p*** |
| --- | --- | --- | --- |
| **FAB Messaging** |  |  |  |
| Dedication (D) – Brilliance (B) | -0.018 | -0.11 | .912 |
| **Gender Majority** |  |  |  |
| FemaleDominated (FD) – MaleDominated (MD) | -0.132 | -0.78 | .433 |
| **Gender Ratio** |  |  |  |
| 75:25 – 60:40 | <−0.001 | -0.01 | .995 |
| 90:10 – 60:40 | -0.144 | -0.88 | .379 |
| **Participant Gender** |  |  |  |
| Female – Male | -0.180 | −1.13 | .259 |
| **Covariates** |  |  |  |
| MCC | -0.156 | -6.16 | **< .001** |
| PM | 0.589 | 23.10 | **< .001** |
| **FAB Messaging × Gender Majority** |  |  |  |
| (D – B) × (FD – MD) | 0.224 | 0.95 | .344 |
| **FAB Messaging × Gender Ratio** |  |  |  |
| (D – B) × (75:25 – 60:40) | 0.074 | 0.32 | .748 |
| (D – B) × (90:10 – 60:40) | 0.049 | 0.21 | .830 |
| **Gender Majority × Gender Ratio** |  |  |  |
| (FD – MD) × (75:25 – 60:40) | -0.154 | -0.68 | .499 |
| (FD – MD) × (90:10 – 60:40) | 0.085 | 0.36 | .716 |
| **FAB Messaging × Participant Gender** |  |  |  |
| (D – B) × (Female – Male) | 0.266 | 1.18 | .237 |
| **Gender Majority × Participant Gender** |  |  |  |
| (FD – MD) × (Female – Male) | 0.039 | 0.17 | .865 |
| **Gender Ratio × Participant Gender** |  |  |  |
| (75:25 – 60:40) × (Female – Male) | -0.214 | -0.96 | .337 |
| (90:10 – 60:40) × (Female – Male) | -0.103 | -0.46 | .648 |
| **FAB Messaging × Gender Majority × Gender Ratio** |  |  |  |
| (D – B) × (FD – MD) × (75:25 – 60:40) | -0.244 | -0.76 | .449 |
| (D – B) × (FD – MD) × (90:10 – 60:40) | -0.254 | -0.78 | .435 |
| **FAB Messaging × Gender Ratio × Participant Gender** |  |  |  |
| (D – B) × (75:25 – 60:40) × (Female – Male) | -0.052 | -0.17 | .868 |
| (D – B) × (90:10 – 60:40) × (Female – Male) | -0.114 | -0.36 | .722 |
| **FAB Messaging × Gender Majority × Participant Gender** |  |  |  |
| (D – B) × (FD – MD) × (Female – Male) | -0.160 | -0.49 | .621 |
| **Gender Majority × Gender Ratio × Participant Gender** |  |  |  |
| (FD – MD) × (75:25 – 60:40) × (Female – Male) | 0.533 | 1.68 | .094 |
| (FD – MD) × (90:10 – 60:40) × (Female – Male) | -0.012 | -0.04 | .970 |
| **FAB messaging × Gender Majority × Gender Ratio × Participant Gender** |  |  |  |
| (D – B) × (FD – MD) × (75:25 – 60:40) × (Female – Male) | -0.078 | -0.17 | .862 |
| (D – B) × (FD – MD) × (90:10 – 60:40) × (Female – Male) | 0.442 | 0.97 | .332 |

*Note.* *β* = standardized coefficient. Reference groups are Brilliance (FAB Messaging), Male Dominated (Gender Majority), 60:40 (Gender Ratio), and Male (Participant Gender).

**Table S22**

Study 4 regression coefficients for the model predicting participants’ belonging (composite score) in the advertised jobs

| **Predictor** | ***β*** | ***t*** | ***p*** |
| --- | --- | --- | --- |
| **FAB Messaging** |  |  |  |
| Dedication (D) – Brilliance (B) | 0.120 | 0.55 | .585 |
| **Gender Majority** |  |  |  |
| FemaleDominated (FD) – MaleDominated (MD) | -0.240 | -1.11 | .266 |
| **Gender Ratio** |  |  |  |
| 75:25 – 60:40 | 0.083 | 0.40 | .688 |
| 90:10 – 60:40 | -0.244 | -1.17 | .244 |
| **Participant Gender** |  |  |  |
| Female – Male | -0.717 | -3.53 | **< .001** |
| **FAB Messaging × Gender Majority** |  |  |  |
| (D – B) × (FD – MD) | 0.138 | 0.45 | .652 |
| **FAB Messaging × Gender Ratio** |  |  |  |
| (D – B) × (75:25 – 60:40) | -0.085 | -0.29 | .773 |
| (D – B) × (90:10 – 60:40) | 0.104 | 0.35 | .724 |
| **Gender Majority × Gender Ratio** |  |  |  |
| (FD – MD) × (75:25 – 60:40) | -0.303 | -1.03 | .302 |
| (FD – MD) × (90:10 – 60:40) | -0.009 | -0.03 | .977 |
| **FAB Messaging × Participant Gender** |  |  |  |
| (D – B) × (Female – Male) | 0.247 | 0.85 | .393 |
| **Gender Majority × Participant Gender** |  |  |  |
| (FD – MD) × (Female – Male) | 0.469 | 1.60 | .110 |
| **Gender Ratio × Participant Gender** |  |  |  |
| (75:25 – 60:40) × (Female – Male) | -0.223 | -0.78 | .434 |
| (90:10 – 60:40) × (Female – Male) | -0.258 | -0.89 | .375 |
| **FAB Messaging × Gender Majority × Gender Ratio** |  |  |  |
| (D – B) × (FD – MD) × (75:25 – 60:40) | 0.232 | 0.56 | .575 |
| (D – B) × (FD – MD) × (90:10 – 60:40) | -0.276 | -0.66 | .508 |
| **FAB Messaging × Gender Ratio × Participant Gender** |  |  |  |
| (D – B) × (75:25 – 60:40) × (Female – Male) | -0.053 | -0.13 | .895 |
| (D – B) × (90:10 – 60:40) × (Female – Male) | -0.071 | -0.17 | .864 |
| **FAB Messaging × Gender Majority × Participant Gender** |  |  |  |
| (D – B) × (FD – MD) × (Female – Male) | 0.081 | 0.20 | .845 |
| **Gender Majority × Gender Ratio × Participant Gender** |  |  |  |
| (FD – MD) × (75:25 – 60:40) × (Female – Male) | 0.837 | 2.05 | **.040** |
| (FD – MD) × (90:10 – 60:40) × (Female – Male) | 1.042 | 2.52 | **.012** |
| **FAB messaging × Gender Majority × Gender Ratio × Participant Gender** |  |  |  |
| (D – B) × (FD – MD) × (75:25 – 60:40) × (Female – Male) | -0.136 | -0.24 | .814 |
| (D – B) × (FD – MD) × (90:10 – 60:40) × (Female – Male) | -0.011 | -0.02 | .985 |

*Note.* *β* = standardized coefficient. Reference groups are Brilliance (FAB Messaging), Male Dominated (Gender Majority), 60:40 (Gender Ratio), and Male (Participant Gender).

**Table S23**

Study 4 regression coefficients, including PM and MCC, for the model predicting participants’ belonging (composite score) in the advertised jobs

| **Predictor** | ***β*** | ***t*** | ***p*** |
| --- | --- | --- | --- |
| **FAB Messaging** |  |  |  |
| Dedication (D) – Brilliance (B) | 0.161 | 1.03 | .304 |
| **Gender Majority** |  |  |  |
| FemaleDominated (FD) – MaleDominated (MD) | 0.073 | 0.47 | .637 |
| **Gender Ratio** |  |  |  |
| 75:25 – 60:40 | 0.086 | 0.58 | .559 |
| 90:10 – 60:40 | 0.237 | 1.57 | .117 |
| **Participant Gender** |  |  |  |
| Female – Male | -0.208 | -1.42 | .156 |
| **Covariates** |  |  |  |
| MCC | -0.431 | -18.52 | **< .001** |
| PM | 0.418 | 17.82 | **< .001** |
| **FAB Messaging × Gender Majority** |  |  |  |
| (D – B) × (FD – MD) | -0.122 | -0.56 | .576 |
| **FAB Messaging × Gender Ratio** |  |  |  |
| (D – B) × (75:25 – 60:40) | -0.132 | -0.63 | .532 |
| (D – B) × (90:10 – 60:40) | -0.344 | -1.62 | .105 |
| **Gender Majority × Gender Ratio** |  |  |  |
| (FD – MD) × (75:25 – 60:40) | -0.315 | -1.50 | .135 |
| (FD – MD) × (90:10 – 60:40) | -0.481 | -2.23 | **.026** |
| **FAB Messaging × Participant Gender** |  |  |  |
| (D – B) × (Female – Male) | -0.106 | -0.51 | .610 |
| **Gender Majority × Participant Gender** |  |  |  |
| (FD – MD) × (Female – Male) | 0.072 | 0.34 | .734 |
| **Gender Ratio × Participant Gender** |  |  |  |
| (75:25 – 60:40) × (Female – Male) | -0.062 | -0.304 | **.**761 |
| (90:10 – 60:40) × (Female – Male) | -0.433 | -2.08 | **.038** |
| **FAB Messaging × Gender Majority × Gender Ratio** |  |  |  |
| (D – B) × (FD – MD) × (75:25 – 60:40) | 0.251 | 0.85 | .398 |
| (D – B) × (FD – MD) × (90:10 – 60:40) | 0.346 | 1.16 | .248 |
| **FAB Messaging × Gender Ratio × Participant Gender** |  |  |  |
| (D – B) × (75:25 – 60:40) × (Female – Male) | 0.058 | 0.20 | .842 |
| (D – B) × (90:10 – 60:40) × (Female – Male) | 0.437 | 1.49 | .138 |
| **FAB Messaging × Gender Majority × Participant Gender** |  |  |  |
| (D – B) × (FD – MD) × (Female – Male) | 0.220 | 0.74 | .461 |
| **Gender Majority × Gender Ratio × Participant Gender** |  |  |  |
| (FD – MD) × (75:25 – 60:40) × (Female – Male) | 0.538 | 1.84 | .067 |
| (FD – MD) × (90:10 – 60:40) × (Female – Male) | 0.872 | 2.93 | **.003** |
| **FAB Messaging × Gender Majority × Gender Ratio × Participant Gender** |  |  |  |
| (D – B) × (FD – MD) × (75:25 – 60:40) × (Female – Male) | -0.272 | -0.65 | .514 |
| (D – B) × (FD – MD) × (90:10 – 60:40) × (Female – Male) | -0.410 | -0.98 | .328 |

*Note.* *β* = standardized coefficient. Reference groups are Brilliance (FAB Messaging), Male Dominated (Gender Majority), 60:40 (Gender Ratio), and Male (Participant Gender).

**Table S24**

Study 4 ANOVA table predicting participants’ anticipated belonging in the jobs using Item 1 of the Belonging measure

| **Measure** | ***df*** | ***M^2^*** | ***F*** | ***p*** | ***η²p f*** |
| --- | --- | --- | --- | --- | --- |
| FAB Messaging | 1 | 51.861 | 12.434 | **< .001** | .013 .115 |
| Gender Majority | 1 | 46.441 | 11.135 | **< .001** | .011 .105 |
| Gender Ratio | 2 | 40.491 | 9.708 | **< .001** | .020 .143 |
| Participant Gender | 1 | 41.173 | 9.872 | **.002** | .010 .101 |
| FAB Messaging × Gender Majority | 1 | 2.298 | 0.551 | .458 | .001 .032 |
| FAB Messaging × Gender Ratio | 2 | 1.466 | 0.352 | .704 | .001 .032 |
| Gender Majority × Gender Ratio | 2 | 4.367 | 1.047 | .351 | .002 .045 |
| FAB Messaging × Participant Gender | 1 | 2.213 | 0.531 | .467 | .001 .032 |
| Gender Majority × Participant Gender | 1 | 592.335 | 142.016 | **< .001** | .128 .383 |
| Gender Ratio × Participant Gender | 2 | 1.161 | 0.278 | .757 | .001 .032 |
| FAB Messaging × Gender Majority × Gender Ratio | 2 | 3.683 | 0.883 | .414 | .002 .045 |
| FAB Messaging × Gender Majority x Participant Gender | 1 | 0.125 | 0.029 | .863 | .000 .000 |
| FAB Messaging × Gender Ratio × Participant Gender | 2 | 0.342 | 0.082 | .921 | .000 .000 |
| Gender Majority × Gender Ratio × Participant Gender | 2 | 60.975 | 14.619 | **< .001** | .029 .173 |
| FAB Messaging × Gender Majority × Gender Ratio × Participant Gender | 2 | 1.072 | 0.257 | .773 | .001 .032 |

**Table S25**

Study 4 ANOVA table predicting participants’ anticipated belonging in the jobs using Item 2 of the Belonging measure

| **Measure** | ***df*** | ***M^2^*** | ***F*** | ***p*** | ***η²p f*** |
| --- | --- | --- | --- | --- | --- |
| FAB Messaging | 1 | 74.815 | 18.455 | **< .001** | .019 .139 |
| Gender Majority | 1 | 62.293 | 15.366 | **< .001** | .016 .128 |
| Gender Ratio | 2 | 1.388 | 0.342 | .710 | .001 .032 |
| Participant Gender | 1 | 26.273 | 6.481 | **.011** | .007 .084 |
| FAB Messaging × Gender Majority | 1 | 4.074 | 1.005 | .316 | .001 .032 |
| FAB Messaging × Gender Ratio | 2 | 4.762 | 1.175 | .309 | .002 .045 |
| Gender Majority × Gender Ratio | 2 | 16.197 | 3.995 | **.019** | .008 .090 |
| FAB Messaging × Participant Gender | 1 | 20.147 | 4.970 | **.026** | .005 .071 |
| Gender Majority × Participant Gender | 1 | 36.117 | 8.909 | **.003** | .009 .095 |
| Gender Ratio × Participant Gender | 2 | 5.587 | 1.378 | .252 | .003 .055 |
| FAB Messaging × Gender Majority × Gender Ratio | 2 | 3.644 | 0.899 | .407 | .002 .045 |
| FAB Messaging × Gender Majority x Participant Gender | 1 | 0.733 | 0.181 | .671 | .000 .000 |
| FAB Messaging × Gender Ratio × Participant Gender | 2 | 1.397 | 0.345 | .708 | .001 .032 |
| Gender Majority × Gender Ratio × Participant Gender | 2 | 1.407 | 0.347 | .707 | .001 .032 |
| FAB Messaging × Gender Majority × Gender Ratio × Participant Gender | 2 | 0.686 | 0.169 | .844 | .000 .000 |

**Table S26**

Study 4 ANCOVA table predicting participants’ belonging (composite measure) in the advertised jobs, with MCC and PM as covariates

| **Measure** | ***df*** | ***M^2^*** | ***F*** | ***p*** | ***η²p f*** |  |
| --- | --- | --- | --- | --- | --- | --- |
| FAB Messaging | 1 | 3.368 | 2.554 | .110 | 0.003 .055 | |
| Gender Majority | 1 | 9.436 | 7.156 | **.008** | 0.007 .084 | |
| Gender Ratio | 2 | 0.412 | 0.312 | .732 | 0.001 .032 | |
| Participant Gender | 1 | 4.027 | 3.054 | .081 | 0.003 .055 | |
| MCC | 1 | 452.117 | 342.832 | **< .001** | 0.263 .597 | |
| PM | 1 | 418.557 | 317.384 | **< .001** | 0.248 .574 | |
| FAB Messaging × Gender Majority | 1 | 0.979 | 0.742 | .389 | 0.001 .032 | |
| FAB Messaging × Gender Ratio | 2 | 0.204 | 0.155 | .857 | 0.000 .000 | |
| Gender Majority × Gender Ratio | 2 | 0.038 | 0.029 | .972 | 0.000 .000 | |
| FAB Messaging × Participant Gender | 1 | 0.564 | 0.427 | .513 | 0.000 .000 | |
| Gender Majority × Participant Gender | 1 | 49.177 | 37.290 | **< .001** | 0.037 .196 | |
| Gender Ratio × Participant Gender | 2 | 1.798 | 1.364 | .256 | 0.003 .055 | |
| FAB Messaging × Gender Majority × Gender Ratio | 2 | 0.338 | 0.256 | .774 | 0.001 .032 | |
| FAB Messaging × Gender Majority × Participant Gender | 1 | 0.002 | 0.002 | .965 | 0.000 .000 | |
| FAB Messaging × Gender Ratio × Participant Gender | 2 | 1.598 | 1.211 | .298 | 0.003 .055 | |
| Gender Majority × Gender Ratio × Participant Gender | 2 | 6.637 | 5.033 | **.007** | 0.010 .101 | |
| FAB Messaging × Gender Majority × Gender Ratio × Participant Gender | 2 | 0.653 | 0.495 | .610 | 0.001 .032 | |

**Table S27**

Study 4 ANCOVA table (MCC and PM as covariates) predicting anticipated belonging using Item 1 of the Belonging measure

| **Measure** | ***df*** | ***M^2^*** | ***F*** | ***p*** | ***η²p f*** |
| --- | --- | --- | --- | --- | --- |
| FAB Messaging | 1 | 1.067 | 0.466 | .495 | .000 .000 |
| Gender Majority | 1 | 13.600 | 5.939 | **.015** | .006 .078 |
| Gender Ratio | 2 | 12.714 | 5.552 | **.004** | .011 .105 |
| Participant Gender | 1 | 4.707 | 2.055 | .152 | .002 .045 |
| MCC | 1 | 158.059 | 69.025 | **< .001** | .067 .268 |
| PM | 1 | 1249.181 | 545.520 | **< .001** | .362 .753 |
| FAB Messaging × Gender Majority | 1 | 0.001 | < .001 | .981 | .000 .000 |
| FAB Messaging × Gender Ratio | 2 | 0.727 | 0.318 | .728 | .001 .032 |
| Gender Majority × Gender Ratio | 2 | 1.741 | 0.760 | .468 | .002 .045 |
| FAB Messaging × Participant Gender | 1 | 0.838 | 0.366 | .545 | .000 .000 |
| Gender Majority × Participant Gender | 1 | 155.784 | 68.031 | **< .001** | .066 .266 |
| Gender Ratio × Participant Gender | 2 | 0.666 | 0.291 | .748 | .001 .032 |
| FAB Messaging × Gender Majority × Gender Ratio | 2 | 0.371 | 0.162 | .850 | .000 .000 |
| FAB Messaging × Gender Majority x Participant Gender | 1 | 2.820 | 1.231 | .267 | .001 .032 |
| FAB Messaging × Gender Ratio × Participant Gender | 2 | 1.105 | 0.483 | .617 | .001 .032 |
| Gender Majority × Gender Ratio × Participant Gender | 2 | 22.291 | 9.743 | **< .001** | .020 .143 |
| FAB Messaging × Gender Majority × Gender Ratio × Participant Gender | 2 | 0.581 | 0.254 | .776 | .001 .032 |

**Table S28**

Study 4 ANCOVA table (MCC and PM as covariates) predicting anticipated belonging using Item 2 of the Belonging measure

| **Measure** | ***df*** | ***M^2^*** | ***F*** | ***p*** | ***η²p f*** |
| --- | --- | --- | --- | --- | --- |
| FAB Messaging | 1 | 6.120 | 2.090 | .149 | .002 .045 |
| Gender Majority | 1 | 5.930 | 2.027 | .155 | .002 .045 |
| Gender Ratio | 2 | 5.90 | 2.015 | .134 | .004 .063 |
| Participant Gender | 1 | 2.790 | 0.954 | .329 | .001 .032 |
| MCC | 1 | 894.810 | 305.986 | **< .001** | .241 .563 |
| PM | 1 | 26.360 | 9.007 | **.003** | .009 .095 |
| FAB Messaging × Gender Majority | 1 | 3.30 | 1.129 | .288 | .001 .032 |
| FAB Messaging × Gender Ratio | 2 | 2.35 | 0.805 | .448 | .002 .045 |
| Gender Majority × Gender Ratio | 2 | 1.75 | 0.599 | .550 | .001 .032 |
| FAB Messaging × Participant Gender | 1 | 5.710 | 1.953 | .163 | .002 .045 |
| Gender Majority × Participant Gender | 1 | 3.070 | 1.050 | .306 | .001 .032 |
| Gender Ratio × Participant Gender | 2 | 4.730 | 1.617 | .199 | .003 .055 |
| FAB Messaging × Gender Majority × Gender Ratio | 2 | 2.250 | 0.768 | .464 | .002 .045 |
| FAB Messaging × Gender Majority x Participant Gender | 1 | 2.620 | 0.895 | .344 | .001 .032 |
| FAB Messaging × Gender Ratio × Participant Gender | 2 | 3.540 | 1.209 | .299 | .003 .055 |
| Gender Majority × Gender Ratio × Participant Gender | 2 | 3.710 | 1.267 | .282 | .003 .055 |
| FAB Messaging × Gender Majority × Gender Ratio × Participant Gender | 2 | 1.790 | 0.613 | .542 | .001 .032 |
